# Association between baseline-stratified trajectory subgroups of serum albumin and outcomes in critically ill patients with early sepsis-associated acute kidney injury: a retrospective study

**DOI:** 10.3389/fmed.2026.1722950

**Published:** 2026-05-29

**Authors:** Qiang Gao, Xinwu Lai, Yu Tian, Tingting Wang, Xiangde Zheng, An Zhang

**Affiliations:** 1 Department of Critical Care Medicine, The Second Affiliated Hospital of Chongqing Medical University, Chongqing, China; 2 Department of Critical Care Medicine, Dazhou Central Hospital, Dazhou, China

**Keywords:** AKI, albumin, outcome, sepsis, trajectory

## Abstract

**Purpose:**

We aimed to explore the association between the serum albumin trajectory following early sepsis-associated acute kidney injury (SA-AKI) and the outcomes of sepsis patients in the intensive care unit (ICU).

**Methods:**

We extracted data of patients with early SA-AKI from the Medical Information Mart for Intensive Care (MIMIC)-IV database. The dynamic levels of albumin within 7 days after AKI onset were used as the exposure factor in this study. The primary outcome was 365-day mortality. Group-based trajectory modeling (GBTM) was used to identify the dynamic characteristics of albumin levels. Kaplan–Meier (KM) survival curves, linear regression and Cox analysis were used to explore the potential associations between different trajectory groups and outcomes.

**Results:**

A total of 1,914 patients with early SA-AKI were included in GBTM. We identified 5 baseline-stratified trajectory subgroups of albumin levels, including four zero-slope trajectories (at levels of 2.0 g/dL, 2.5 g/dL, 3.0 g/dL and 3.5 g/dL) and a slightly ascending trajectory (at a level of >4.1 g/dL). Compared with the steady-state trajectory at a level of 3.0 g/dL, patients with the steady-state trajectory at a level of 2.0 g/dL had higher 30-, 90-, 180-, and 365-day mortality risk and longer hospital length of stay (LOS). And patients with the trajectory at a level of >4.1 g/dL had higher 365-day mortality risk. Moreover, subgroup analysis indicated that the association between serum albumin trajectories and mortality risk interacted with age and sex. Further sensitivity analysis confirmed the robustness of these results through similar trends.

**Conclusion:**

The baseline-stratified trajectory subgroups of serum albumin following early SA-AKI were associated with both short- and long-term mortality and hospital LOS in critically ill patients with sepsis, which can be used as a valuable indicator for guiding risk stratification and predicting prognosis in such patients.

## Introduction

Sepsis, a worldwide public health dilemma, has a high in-hospital mortality rate that ranges from 15.0 to 26.7% (1–4). A dysregulated host response to infection in sepsis patients leads to organ dysfunction (5), often resulting in sepsis-associated acute kidney injury (SA-AKI). SA-AKI occurs in approximately one sixth of intensive care unit (ICU) patients according to the latest consensus report (6, 7) and in more than 40% of sepsis patients in the ICU (8–10). SA-AKI is significantly associated with poor clinical outcomes, including prolonged hospitalization, increased medical expenses and an unacceptable in-hospital mortality rate as high as 18.0–42.2% (6, 8, 10). Therefore, it is important to explore potential factors associated with the outcomes of SA-AKI for early risk identification, stratification and even intervention.

Albumin is an important component of serum and plays an important role in maintaining plasma colloid osmotic pressure and other physiological processes, such as immune regulation, matter transportation and antioxidation (11). Hypoalbuminemia is a common complication of sepsis and is associated with a high mortality risk in patients with sepsis (12, 13). In addition, some studies have confirmed that higher serum albumin levels indicate better prognosis in patients with AKI (14, 15). However, whether there is a similar correlation between serum albumin levels and outcomes in patients with SA-AKI remains uncertain, especially when early administration of albumin has been shown to increase the risk of SA-AKI in patients with sepsis (16).

Given that early SA-AKI, which occurs within 48 h after sepsis, is the dominant form in patients with SA-AKI (17), we focused on early SA-AKI to further decrease the heterogeneity of the research population. Thus, we extracted the records of early SA-AKI patients from the Medical Information Mart for Intensive Care (MIMIC)-IV database. We aimed to investigate the potential association between the dynamic levels of serum albumin following early SA-AKI and outcomes in ICU patients with sepsis. We hope to provide reasonable evidence to support the individualized clinical decision-making of this special group.

## Methods

### Patients

The data analyzed in this retrospective study were extracted from the MIMIC-IV database, version 3.1, documenting comprehensive records such as patient demographics, laboratory findings, vital signs, disease diagnosis, and follow-up survival status from 2008 to 2022. The MIMIC-IV v3.1 database, a freely accessible dataset of electronic health records, contains more than 94,000 ICU admissions at the Beth Israel Deaconess Medical Center in Boston, Massachusetts, USA, within the aforementioned timeframe (18–20). The author, Qiang Gao, has completed the online courses and obtained access to the database (Certification number: 64760930). The study adhered to the Strengthening the Reporting of Observational Studies in Epidemiology (STROBE) statement for observational studies (21).

The ICU-admitted patients with AKI were selected. If the same patient had multiple ICU admissions, only the initial ICU admission would be extracted. The AKI was defined according to the Kidney Disease: Improving Global Outcomes (KDIGO) guidelines (22). The definition of sepsis was consistent with the Sepsis 3.0 criteria (5), including a suspicion of infection and a Sequential Organ Failure Assessment (SOFA) score ≥ 2. The diagnosis of early SA-AKI was based on the consensus report of the 28th Acute Disease Quality Initiative workgroup (7), which defines SA-AKI as AKI occurring within 48 h following the onset of sepsis. We extracted the occurrence times of sepsis and AKI and then calculated time interval between the two onset times to determine SA-AKI. The inclusion criteria were as follows: (1) patients with AKI diagnosed within 48 h after ICU admission and (2) patients whose serum albumin was recorded at least 3 times within 1 week after SA-AKI onset. The exclusion criteria were as follows: (1) patients whose age was <18 years, (2) patients whose length of ICU stay was <24 h, (3) patients whose death occurred within 8 days after AKI and (4) patients whose serum albumin levels were recorded < 3 times.

### Data extraction

We employed Structured Query Language (SQL) via pgAdmin IV (Version 7.5) to retrieve detailed records from the MIMIC-IV database. The dataset included demographic details, initial vital signs and laboratory findings after admission to the ICU, serum albumin records from the second day to the eighth day after AKI onset, comorbidities, SOFA score, Simplified Acute Physiology Score II (SAPS II), Charlson comorbidity index (CCI), time records of AKI and sepsis occurrence, start times of invasive mechanical ventilation (IMV) and renal replacement therapy (RRT), duration of IMV, RRT days, support of albumin, vasopressor and nutrition from the second day to the eighth day after AKI onset and outcomes.

### Outcomes

In this study, the time of SA-AKI onset was defined as the time zero. The primary outcome of this study was 365-day all-cause mortality following AKI onset, with secondary outcome data including 30-day, 90-day and 180-day all-cause mortality following AKI onset, durations of IMV after AKI, number of RRT days after AKI, ICU length of stay (LOS) after AKI, and hospital LOS.

### Statistical analysis

Group-based trajectory modeling (GBTM) was employed to determine the dynamic changes in serum albumin concentration over time via SAS 9.4 (TS Level 1M7 for Windows). The trajectory models were fitted from two groups to six groups. Each trajectory was sequentially modeled using a third-order (cubic), second-order (quadratic), and first-order (linear) function. The model’s suitability was assessed on the basis of the following criteria: (1) values of Bayesian information criterion (BIC) and Akaike information criterion (AIC) closer to 0, (2) average posterior probability (AvePP) per group > 0.7, (3) odds of correct classification (OCC) > 5, (4) proportion per group > 5%, and (5) the actual clinical situation and interpretability.

Additional data analyses were performed using SPSS 27.0 software (SPSS Inc., Chicago, IL, United States). Variables with missing values exceeding 20% were excluded from statistical analysis. The missing values of continuous variables in this study were replaced by multiple-imputation method. Then 5 data sets were generated. Regression analysis results of all data sets including the raw data set were pooled into one result by SPSS. The Shapiro–Wilk test was employed to assess the normality of continuous variables. For data with a skewed distribution, the median and interquartile range (IQR) were reported, and comparisons were conducted using the Kruskal-Wallis H-test. Conversely, data conforming to a normal distribution were summarized as the mean ± standard deviation (SD), and comparisons were conducted using the one-way ANOVA. For categorical variables, the Chi-square test or Fisher’s exact test was applied to determine significant differences.

Moreover, Kaplan–Meier (KM) survival curve was constructed to illustrate the variations in survival status across different trajectory groups over the follow-up time. The statistical significance of the differences observed in the KM survival analysis was assessed using the log-rank test. Linear regression and Cox analysis were used to explore the potential associations between different trajectory groups and outcomes. A two-sided *p* < 0.05 was considered to indicate statistical significance.

## Results

### Trajectory analysis results

**Figure 1:**
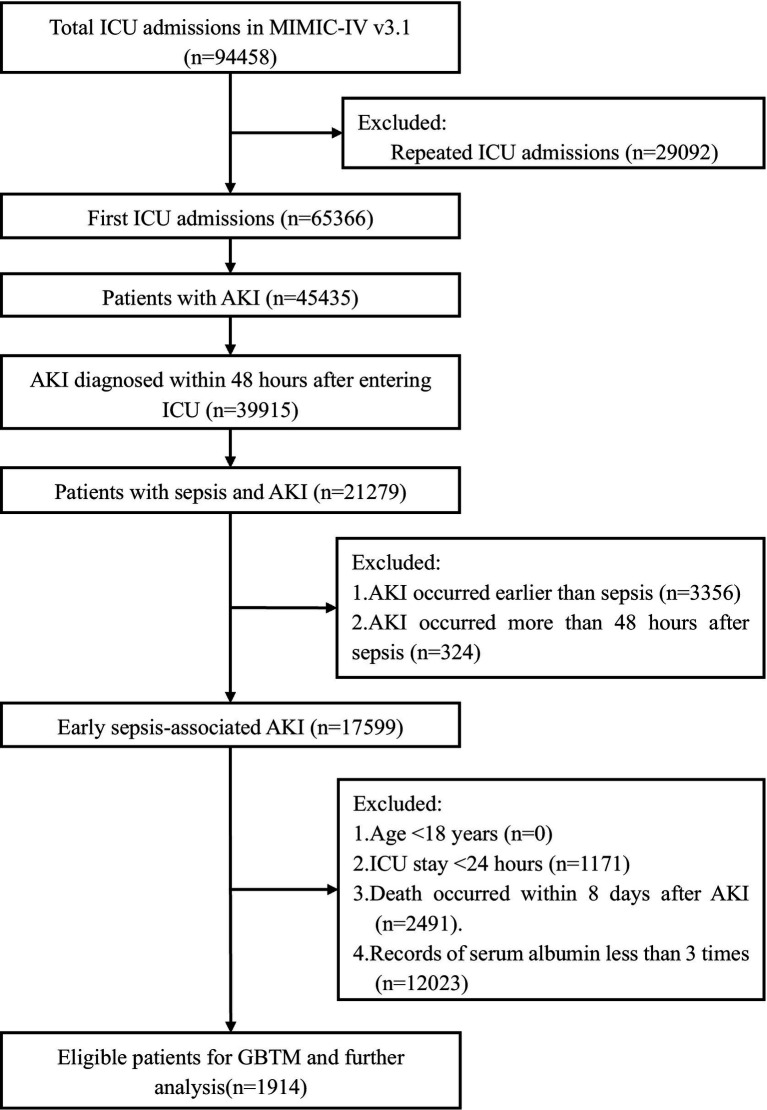
Flow chart of patient enrolment. ICU, Intensive Care Unit; AKI, acute kidney injury; GBTM, group-based trajectory modeling.

**Figure 2:**
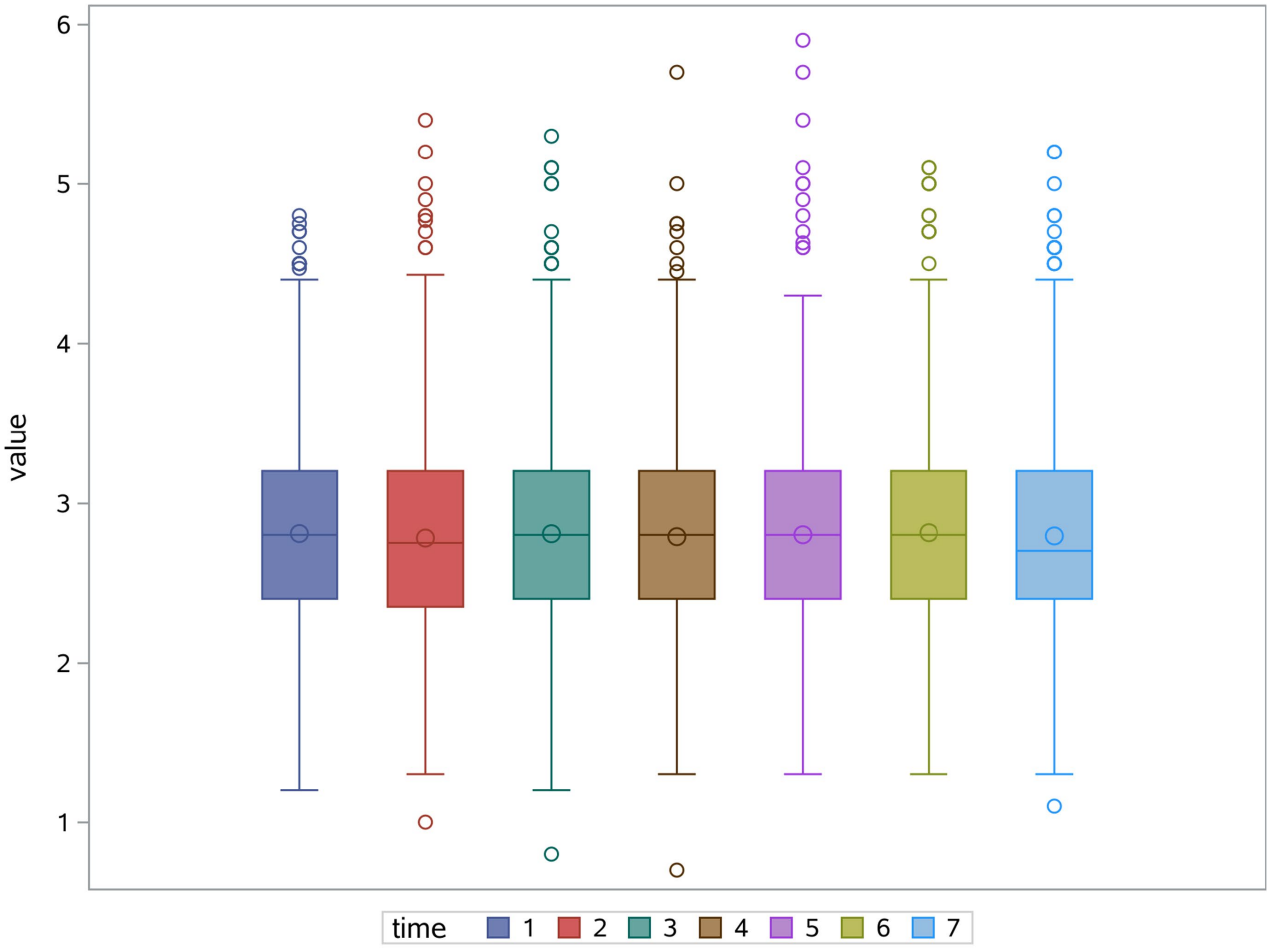
Distribution characteristics of serum albumin values within 7 days after SA-AKI onset in 1,914 patients. The rectangle represents the interquartile range. The horizontal line in the rectangle represents the median. The circle in the rectangle represents the mean value. The remaining circles represent some scattered extreme values.

**Figure 3:**
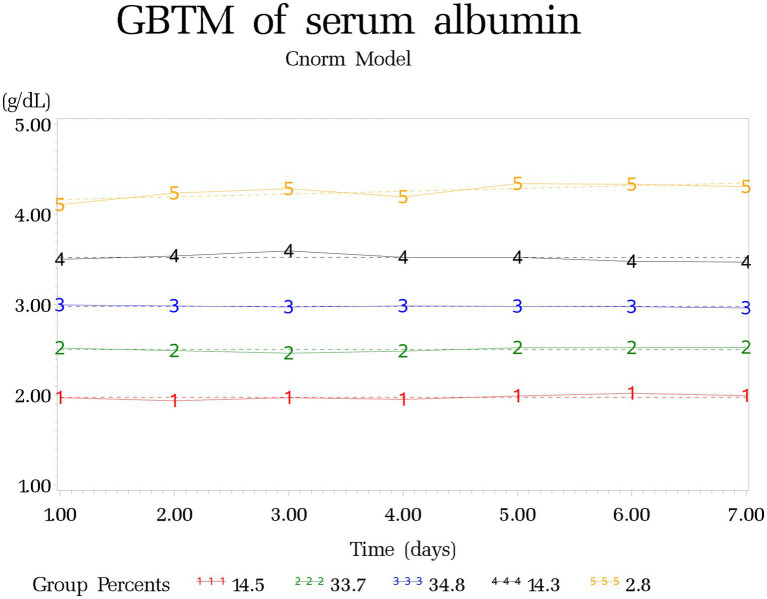
Trajectories of serum albumin. GBTM, group-based trajectory modeling. The solid line represents the actual trend. The dotted line represents the predicted trend.

In this study, a total of 1,914 patients diagnosed with early SA-AKI were included in the GBTM analysis. The screening process is illustrated in Figure 1. The distribution of serum albumin values within 7 days after AKI onset is demonstrated in Figure 2. The details of the missing data are provided in Supplementary Table S1. We fitted five trajectory models from two groups to six groups. The estimation of the parameters for the five models is presented in Supplementary Table S2. Supplementary Table S3 displays the performance of the five trajectory models. In accordance with our evaluation criteria, we eventually chose the 5–0,0,0,0,1 model as the most ideal model, including four zero-slope trajectories (at levels of 2.0 g/dL, 2.5 g/dL, 3.0 g/dL and 3.5 g/dL) and a slightly ascending trajectory (at a level of >4.1 g/dL) (Figure 3; Supplementary Table S2). The distribution of albumin values within each trajectory group is presented in Supplementary Figures S1–S5. Although the proportion of one trajectory group in this model is less than 5%, actual quantity of this trajectory group is sufficient for further statistical analysis. In addition, our subsequent baseline analysis also proved the unique clinical features of this group. And keeping this group separate can prevent its extreme values from affecting other trajectories to some extent and increase the interpretability of the results.

### Baseline characteristics and outcomes

Baseline characteristics of original data set is presented in Table 1. The median age of all sepsis patients with early SA-AKI was 63 years (IQR, 53–74; range, 18–98 years), and the males constituted 59.8% of the patient group. Comparisons of the baseline and clinical features revealed differences in many variables among the five groups. Notably, patients in Group 1 had the highest median age, SAPS II, ICU LOS after AKI, and hospital LOS after AKI, as well as the highest proportion of malignancy, enteral nutrition support, and IMV after AKI. Patients in Group 5 had the highest median total bilirubin, urea nitrogen, creatinine, prothrombin time (PT), partial thromboplastin time (PTT) and amount of albumin infusion, and the highest proportion of MICU admission and cirrhosis. And this group also had the lowest median platelet count, hemoglobin, alanine aminotransferase (ALT) and aspartate aminotransferase (AST), and the lowest proportion of malignancy, chronic obstructive pulmonary disease (COPD), acute pancreatitis and IMV after AKI. Moreover, the Kruskal-Wallis H-test results of variables with missing data after multiple interpolation maintained a consistent trend compared with those before interpolation (Supplementary Table S4).

**Table 1:** Comparison of baseline and clinical characteristics between the 5 trajectory groups.

Variables	Total (*n* = 1,914)	Group 1 (*n* = 268)	Group 2 (*n* = 658)	Group 3 (*n* = 679)	Group 4 (*n* = 255)	Group 5 (*n* = 54)	*p*
Age, years	63 (53,74)	66 (54,75)	64 (53,76)	62 (53,73)	61 (52,70)	61 (55,69)	0.008
Male, *n* (%)	1,144 (59.8)	153 (57.1)	371 (56.4)	426 (62.7)	158 (62.0)	36 (66.7)	0.091
Race, *n* (%)							0.888
White	1,184 (61.9)	165 (61.6)	414 (62.9)	413 (60.8)	156 (61.2)	36 (66.7)	
Black	179 (9.4)	30 (11.2)	58 (8.8)	64 (9.4)	22 (8.6)	5 (9.3)	
Hispanic	71 (3.7)	6 (2.2)	26 (4.0)	25 (3.7)	12 (4.7)	2 (3.7)	
Asian	56 (2.9)	12 (4.5)	21 (3.2)	16 (2.4)	6 (2.4)	1 (1.9)	
Other	424 (22.2)	55 (20.5)	139 (21.1)	161 (23.7)	59 (23.1)	10 (18.5)	
ICU type, *n* (%)							<0.001
MICU	718 (37.5)	95 (35.4)	216 (32.8)	268 (39.5)	108 (42.4)	31 (57.4)	
SICU	529 (27.6)	77 (28.7)	168 (25.5)	210 (30.9)	61 (24.0)	13 (24.1)	
MICU/SICU	328 (17.1)	72 (26.9)	159 (24.2)	77 (11.3)	18 (7.1)	2 (3.7)	
CVICU	159 (8.3)	14 (5.2)	55 (8.4)	57 (8.4)	29 (11.4)	4 (7.4)	
CCU	140 (7.3)	5 (1.9)	48 (7.3)	56 (8.2)	29 (11.4)	2 (3.7)	
Other	40 (2.1)	5 (1.9)	12 (1.8)	11 (1.6)	10 (3.9)	2 (3.7)	
Score system							
SOFA score	8 (6, 11)	8 (6, 11)	8 (5, 11)	8 (6, 11)	8 (6, 11)	10 (8, 11)	0.139
SAPS II	44 (36, 55)	49 (37, 60)	46 (37, 57)	43 (35, 53)	41 (33, 51)	46 (39, 54)	<0.001
CCI	6 (4, 7)	6 (3, 8)	6 (4, 8)	5 (3, 7)	5 (4, 7)	6 (4, 8)	0.264
Comorbidities, *n* (%)							
Pneumonia	798 (41.7)	117 (43.7)	294 (44.7)	263 (38.7)	103 (40.4)	21 (38.9)	0.228
Hypertension	629 (32.9)	100 (37.3)	212 (32.2)	222 (32.7)	79 (31.0)	16 (29.6)	0.524
Diabetes	611 (31.9)	94 (35.1)	209 (31.7)	212 (31.2)	82 (32.2)	14 (25.9)	0.683
Cirrhosis	607 (31.7)	42 (15.7)	152 (23.1)	252 (37.1)	120 (47.1)	41 (75.9)	<0.001
Hyperlipoidemia	531 (27.7)	81 (30.2)	184 (28.0)	178 (26.2)	74 (29.0)	14 (25.9)	0.749
CKD	436 (22.8)	55 (20.5)	161 (24.5)	146 (21.5)	63 (24.7)	11 (20.4)	0.527
Malignancy	342 (17.9)	71 (26.5)	140 (21.3)	102 (15.0)	25 (9.8)	4 (7.4)	<0.001
COPD	174 (9.1)	20 (7.5)	56 (8.5)	58 (8.5)	37 (14.5)	3 (5.6)	0.024
Myocardial infarction	172 (9.0)	22 (8.2)	59 (9.0)	73 (10.8)	14 (5.5)	4 (7.4)	0.149
Stroke	137 (7.2)	23 (8.6)	48 (7.3)	43 (6.3)	21 (8.2)	2 (3.7)	0.567
Acute pancreatitis	113 (5.9)	26 (9.7)	52 (7.9)	25 (3.7)	10 (3.9)	0 (0.0)	<0.001
Vital signs							
Heart rate, bpm	94 (80, 109)	100 (87, 115)	96 (80, 112)	90 (80, 105)	90 (77, 103)	93 (76, 103)	<0.001
NMAP, mmHg	78 (67, 91)	78 (66, 89)	76 (65, 89)	79 (69, 93)	80 (69, 94)	74 (62, 84)	<0.001
Respiratory rate, bpm	20 (16, 24)	21 (18, 26)	19 (16, 25)	20 (16, 24)	19 (16, 25)	18 (16, 23)	<0.001
SpO_2_, %	98 (95, 100)	97 (95, 100)	97 (94, 100)	98 (95, 100)	98 (95, 100)	98 (96, 100)	0.006
Laboratory findings							
WBC, 10^9^/L	11.7 (7.5, 17.5)	12.5 (7.6, 20.4)	12.6 (8.1, 18.8)	10.9 (7.3, 16.2)	11.0 (7.0, 16.0)	9.9 (7.2, 14.1)	<0.001
Platelet, 10^9^/L	143 (87, 230)	167 (110, 281)	158 (92, 242)	136 (83, 220)	126 (76, 191)	90 (65, 147)	<0.001
Hemoglobin, g/dL	9.9 (8.3, 11.5)	9.4 (8.1, 10.9)	9.9 (8.5, 11.6)	10.0 (8.5, 11.5)	9.6 (8.0, 11.8)	9.2 (7.5, 11.2)	0.013
Total bilirubin, mg/dL	1.3 (0.6, 3.9)	1.0 (0.5, 2.4)	1.2 (0.5, 3.2)	1.4 (0.6, 4.4)	1.7 (0.7, 5.2)	3.4 (1.6, 11.5)	<0.001
ALT, IU/L	38 (20, 112)	32 (18, 83)	41 (20, 119)	43 (20, 143)	34 (19, 93)	28 (17, 54)	0.002
AST, IU/L	67 (33, 181)	54 (28, 123)	71 (33, 179)	73.5 (36, 225)	59 (35, 168)	51 (34, 109)	0.002
Urea nitrogen, mg/dL	26 (16, 44)	30 (18, 47)	26 (16, 43)	24 (15, 41)	27 (15, 47)	32 (21, 48)	0.009
Creatinine, mg/dL	1.3 (0.8, 2.2)	1.3 (0.8, 2.0)	1.3 (0.8, 2.2)	1.2 (0.8, 2.2)	1.4 (0.9, 2.2)	1.8 (1.1, 3.0)	0.029
PT, s	16.6 (14.0, 21.1)	15.9 (14.1, 18.9)	16.1 (13.8, 19.8)	16.9 (13.8, 21.7)	17.9 (14.3, 24.3)	21.1 (15.8, 26.9)	<0.001
PTT, s	34.3 (29.3, 44.2)	33.6 (29.3, 43.4)	33.8 (29.0, 42.3)	34.6 (29.2, 45.0)	34.7 (30.0, 46.7)	42.8 (32.7, 55.1)	0.004
Sodium, mEq/L	138 (134, 141)	138 (134, 142)	138 (134, 142)	138 (134, 141)	137 (134, 141)	136 (132, 140)	0.049
Potassium, mEq/L	4.2 (3.7, 4.7)	4.1 (3.7, 4.6)	4.2 (3.7, 4.7)	4.2 (3.8, 4.9)	4.2 (3.7, 4.6)	4.0 (3.6, 4.8)	0.110
Total calcium, mg/dL	8.1 (7.6, 8.7)	7.7 (7.2, 8.2)	8.0 (7.4, 8.6)	8.3 (7.8, 8.8)	8.5 (8.0, 8.9)	8.6 (8.0, 9.4)	<0.001
Chloride, mEq/L	103 (99, 108)	104 (99, 109)	104 (99, 108)	103 (99, 107)	102 (97, 107)	101 (95, 107)	<0.001
Glucose, mg/dL	136 (107, 183)	142 (103, 188)	137 (110, 185)	137 (108, 186)	131 (106, 177)	113 (96, 142)	0.006
Anion gap, mEq/L	15 (12, 19)	15 (12, 18)	15 (13, 18)	15 (12, 19)	15 (12, 19)	18 (14, 21)	0.058
pH	7.35 (7.27, 7.41)	7.35 (7.25, 7.41)	7.34 (7.25, 7.41)	7.35 (7.29, 7.41)	7.37 (7.30, 7.41)	7.38 (7.31, 7.43)	<0.001
PCO_2_, mmHg	40 (33, 47)	38 (33, 47)	40 (34, 48)	41 (34, 47)	39 (34, 48)	35 (28, 43)	<0.001
PO_2_, mmHg	85 (49, 158)	76 (48, 136)	86 (49, 157)	88 (50, 178)	80 (48, 154)	89 (55, 141)	0.404
Lactate, mmol/L	2.3 (1.5, 3.7)	2.5 (1.6, 3.6)	2.3 (1.5, 4.0)	2.2 (1.5, 3.7)	2.1 (1.5, 3.6)	2.5 (1.9, 3.5)	0.389
^*^Albumin infusion, g	0 (0, 38)	0 (0, 25)	0 (0, 25)	0 (0, 38)	0 (0, 75)	119 (0, 208)	<0.001
^*^Vasoactive support							
Dobutamine, mg	0 (0, 0)	0 (0, 0)	0 (0, 0)	0 (0, 0)	0 (0, 0)	0 (0, 0)	0.991
Dopamine, mg	0 (0, 0)	0 (0, 0)	0 (0, 0)	0 (0, 0)	0 (0, 0)	0 (0, 0)	0.151
Epinephrine, mg	0 (0, 0)	0 (0, 0)	0 (0, 0)	0 (0, 0)	0 (0, 0)	0 (0, 0)	0.554
Norepinephrine, mg	0 (0, 3.0)	0 (0, 18.0)	0 (0, 4.6)	0 (0, 0.3)	0 (0, 0)	0 (0, 7.6)	<0.001
Phenylephrine, mg	0 (0, 0)	0 (0, 0)	0 (0, 0)	0 (0, 0)	0 (0, 0)	0 (0, 0)	0.057
Vasopressin, unit	0 (0, 0)	0 (0, 0.2)	0 (0, 0)	0 (0, 0)	0 (0, 0)	0 (0, 0)	<0.001
^*^Nutritional support, *n* (%)							
Enteral nutrition	121 (6.3)	32 (11.9)	51 (7.8)	33 (4.9)	3 (1.2)	2 (3.7)	<0.001
Parenteral nutrition	677 (35.4)	113 (42.2)	233 (35.4)	235 (34.6)	77 (30.2)	19 (35.2)	0.073
IMV after AKI	1,151 (60.1)	169 (63.1)	405 (61.6)	417 (61.4)	141 (55.3)	19 (35.2)	<0.001
RRT after AKI	360 (18.8)	57 (21.3)	134 (20.4)	117 (17.2)	38 (14.9)	14 (25.9)	0.109
ICU LOS after AKI, d	5.7 (2.5, 11.7)	8.5 (2.7, 14.3)	6.7 (2.8, 12.9)	5.0 (2.6, 10.2)	4.2 (2.0, 9.6)	5.0 (2.1, 11.4)	<0.001
Hospital LOS after AKI, d	15.8 (10.5, 25.1)	18.3 (12.7, 30.4)	17.0 (10.8, 25.9)	14.7 (9.7, 22.8)	13.4 (9.5, 22.2)	17.4 (10.8, 32.3)	<0.001
Mortality, *n* (%)							
30-day	461 (24.1)	91 (34.0)	162 (24.6)	134 (19.7)	57 (22.4)	17 (31.5)	<0.001
90-day	664 (34.7)	131 (48.9)	237 (36.0)	191 (28.1)	76 (29.8)	29 (53.7)	<0.001
180-day	744 (38.9)	142 (53.0)	263 (40.0)	221 (32.5)	87 (34.1)	31 (57.4)	<0.001
365-day	832 (43.5)	152 (56.7)	291 (44.2)	250 (36.8)	103 (40.4)	36 (66.7)	<0.001

ICU, intensive care unit; MICU, medical intensive care unit; SICU, surgical intensive care unit; MICU/SICU, medical/surgical intensive care unit; CVICU, cardiac vascular intensive care unit; CCU, coronary care unit; SOFA, Sequential Organ Failure Assessment; SAPS, simplified acute physiology score; CCI, Charlson comorbidity index; CKD, chronic kidney disease; COPD, chronic obstructive pulmonary disease; NMAP, non-invasive mean arterial pressure; SpO_2_, peripheral oxygen saturation; WBC, white blood cell count; ALT, alanine aminotransferase; AST, aspartate aminotransferase; PT, prothrombin time; PTT, partial thromboplastin time; RRT, renal replacement therapy; IMV, invasive mechanical ventilation; LOS, length of stay. ^*^The statistical window of albumin dosing, vasoactive support and nutritional support was consistent with the exposure window of serum trajectory. Group 1, 2.0 g/dL; Group 2, 2.5 g/dL; Group 3, 3.0 g/dL; Group 4, 3.5 g/dL; Group 5, >4.1 g/dL.

**Figure 4:**
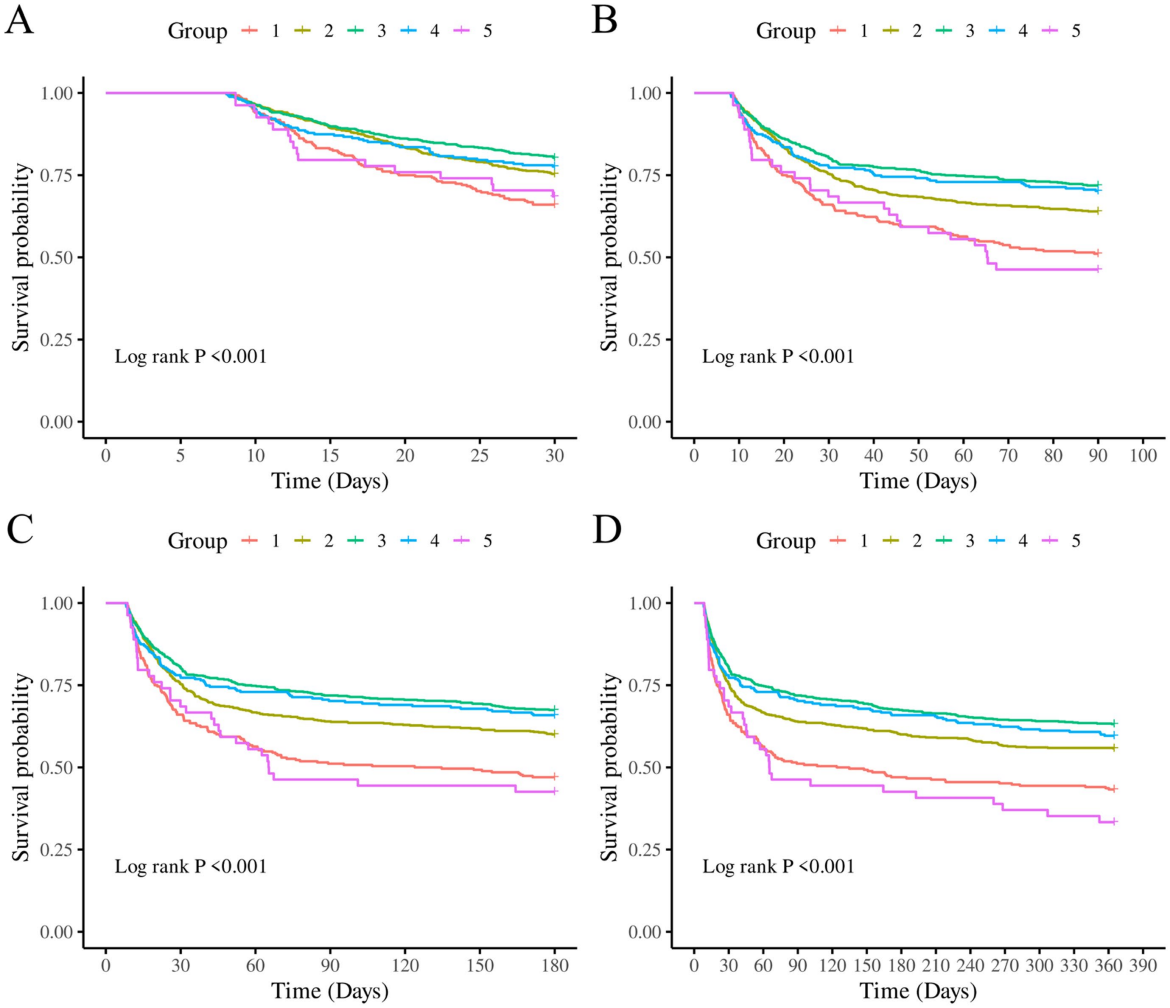
Kaplan–Meier survival curves for 30-day **(A)**, 90-day **(B)**, 180-day **(C)**, and 365-day **(D)** mortality. Group 1, 2.0 g/dL; Group 2, 2.5 g/dL; Group 3, 3.0 g/dL; Group 4, 3.5 g/dL; Group 5, >4.1 g/dL.

The 30-, 90-, 180-, and 365-day mortality rates among the 1,914 patients were 24.1, 34.7, 38.9, and 43.5%, respectively. Patients in Group 3 had the lowest 30-, 90-, 180-, and 365-day mortality rates among the five groups. Furthermore, patients in Group 1 had the highest 30-day mortality rate, and patients in Group 5 had the highest 90-, 180-, and 365-day mortality rates. Interestingly, the KM survival curves revealed that patients in Group 3 always maintained the lowest mortality rates within 365 days (Figure 4).

### Correlation analysis

According to the baseline differences observed among the five groups, the following variables were included as covariates to adjust subsequent regression analyses: age, sex, ICU type, SAPS II, cirrhosis, malignancy, COPD, acute pancreatitis, heart rate, non-invasive mean arterial pressure (NMAP), respiratory rate, SpO_2_, white blood cell count (WBC), platelet count, hemoglobin, total bilirubin, ALT, AST, urea nitrogen, creatinine, PT, PTT, sodium, total calcium, chloride, glucose, anion gap, pH, PCO_2_, albumin infusion, norepinephrine, phenylephrine, vasopressin, enteral nutrition, parenteral nutrition, IMV after AKI.

As shown in Table 2, the results of the multivariate Cox regression revealed that Group 1 exhibited higher 30-, 90-, 180-, and 365-day mortality risk, with Group 3 being used as a reference. Specifically, the hazard ratios (HRs) and corresponding 95% confidence intervals (CIs) were as follows: 30-day mortality risk (HR 1.89, 95% CI 1.41–2.53, *p* < 0.001), 90-day mortality risk (HR 2.00, 95% CI 1.57–2.55, *p* < 0.001), 180-day mortality risk (HR 1.94, 95% CI 1.55–2.45, *p* < 0.001), and 365-day mortality (HR 1.86, 95% CI 1.50–2.32, *p* < 0.001). Group 2 demonstrated higher 90-, 180-, and 365-day mortality risk (HR 1.28, 95% CI 1.04–1.56, *p* = 0.018; HR 1.26, 95% CI 1.04–1.52, *p* = 0.016; HR 1.23, 95% CI 1.14–1.47, *p* = 0.022; respectively). And Group 5 was associated with elevated 365-day mortality risk (HR 1.51, 95% CI 1.03–2.20, *p* = 0.035).

**Table 2:** Cox regression analysis for the association between trajectory groups and mortalities.

Mortalities	Unadjusted		Model I		Model II		Model III	
	HR (95% CI)	*p*	HR (95% CI)	*p*	HR (95% CI)	*p*	HR (95% CI)	*p*
30-day mortality								
Group 1	1.88 (1.44–2.46)	<0.001	1.84 (1.41–2.40)	<0.001	2.03 (1.52–2.70)	<0.001	1.89 (1.41–2.53)	<0.001
Group 2	1.27 (1.01–1.60)	0.040	1.23 (0.98–1.55)	0.077	1.23 (0.97–1.57)	0.092	1.22 (0.96–1.56)	0.104
Group 3	1.00 (Reference)		1.00 (Reference)		1.00 (Reference)		1.00 (Reference)	
Group 4	1.17 (0.86–1.59)	0.330	1.20 (0.88–1.64)	0.252	1.22 (0.88–1.67)	0.229	1.16 (0.84–1.61)	0.359
Group 5	1.75 (1.06–2.90)	0.030	1.77 (1.07–2.94)	0.026	1.26 (0.75–2.11)	0.380	1.15 (0.68–1.97)	0.600
90-day mortality								
Group 1	2.00 (1.60–2.50)	<0.001	1.98 (1.58–2.47)	<0.001	2.13 (1.68–2.71)	<0.001	2.00 (1.57–2.55)	<0.001
Group 2	1.33 (1.11–1.62)	0.003	1.30 (1.07–1.57)	0.007	1.29 (1.06–1.58)	0.011	1.28 (1.04–1.56)	0.018
Group 3	1.00 (Reference)		1.00 (Reference)		1.00 (Reference)		1.00 (Reference)	
Group 4	1.09 (0.84–1.42)	0.530	1.12 (0.86–1.46)	0.420	1.14 (0.87–1.50)	0.333	1.12 (0.85–1.47)	0.438
Group 5	2.20 (1.49–3.25)	<0.001	2.25 (1.52–3.33)	<0.001	1.63 (1.09–2.44)	0.017	1.49 (0.97–2.27)	0.066
180-day mortality								
Group 1	1.91 (1.55–2.36)	<0.001	1.90 (1.54–2.35)	<0.001	2.06 (1.64–2.59)	<0.001	1.94 (1.55–2.45)	<0.001
Group 2	1.29 (1.08–1.55)	0.005	1.26 (1.05–1.51)	0.041	1.28 (1.06–1.54)	0.011	1.26 (1.04–1.52)	0.016
Group 3	1.00 (Reference)		1.00 (Reference)		1.00 (Reference)		1.00 (Reference)	
Group 4	1.08 (0.84–1.38)	0.559	1.10 (0.86–1.42)	0.433	1.13 (0.88–1.46)	0.333	1.11 (0.86–1.43)	0.428
Group 5	2.10 (1.44–3.06)	<0.001	2.16 (1.48–3.14)	<0.001	1.59 (1.08–2.33)	0.020	1.43 (0.95–2.15)	0.085
365-day mortality								
Group 1	1.85 (1.51–2.26)	<0.001	1.85 (1.51–2.26)	<0.001	1.96 (1.58–2.43)	<0.001	1.86 (1.50–2.32)	<0.001
Group 2	1.27 (1.08–1.51)	0.005	1.25 (1.05–1.47)	0.011	1.24 (1.04–1.48)	0.016	1.23 (1.14–1.47)	0.022
Group 3	1.00 (Reference)		1.00 (Reference)		1.00 (Reference)		1.00 (Reference)	
Group 4	1.13 (0.90–1.42)	0.306	1.15 (0.92–1.45)	0.221	1.20 (0.95–1.51)	0.138	1.17 (0.92–1.48)	0.196
Group 5	2.23 (1.57–3.16)	<0.001	2.29 (1.61–3.25)	<0.001	1.69 (1.18–2.43)	0.004	1.51 (1.03–2.20)	0.035

HR, hazard ratio; CI, confidence interval. Model I = Adjusted for age and sex; Model II = Model I + (ICU type, SAPS II, cirrhosis, malignancy, COPD, acute pancreatitis, heart rate, NMAP, respiratory rate, SpO2, WBC, platelet, hemoglobin, total bilirubin, ALT, AST, urea nitrogen, creatinine, PT, PTT, sodium, total calcium, chloride, glucose, anion gap, pH, PCO2); Model III = Model II + (albumin infusion, norepinephrine, phenylephrine, vasopressin, enteral nutrition, parenteral nutrition, IMV after AKI).

Group 1, level of 2.0 g/dL; Group 2, level of 2.5 g/dL; Group 3, level of 3.0 g/dL; Group 4, level of 3.5 g/dL; Group 5, level of >4.1 g/dL.

Additionally, univariate linear regression analysis revealed that both Group 1 and Group 2 were associated with extended ICU LOS, increased days of RRT and prolonged durations of IMV (Supplementary Table S5). However, these significances disappeared after adjusting covariates. When Group 4 and Group 5 were compared with Group 3, no statistically significant difference was observed in terms of the association with the ICU LOS, number of RRT days, or duration of IMV. More importantly, both Group 1 and Group 5 were significantly associated with prolonged hospital LOS even after adjusting the covariates (*β* 3.36, 95% CI 1.76–6.70, *p* < 0.001; *β* 3.47, 95% CI 3.64–13.09, *p* < 0.001; respectively).

### Subgroup analysis

In the subgroup analysis, we employed Group 3 as a reference and divided the trajectory groups into multiple binary categories for comparative analysis to better clarify the results (Supplementary Tables S6–S9). The subgroup analysis indicated consistent relationships between albumin trajectories and both 180-day and 365-day mortality across all subgroups (all *p* > 0.05 for the interaction). Whereas, the association between some albumin trajectories and short-term mortality significantly interacted with age and sex. In the subgroup of patients aged <65 years, Group 4 had significantly increased 30-day mortality risk (HR 1.61, 95% CI 1.05–2.47, *p* = 0.031), and Group 5 had significantly increased 90-day mortality risk (HR 2.12, 95% CI 1.22–3.67, *p* = 0.008). Afterwards, the interaction effects disappeared with increasing follow-up time. In addition, the 30-day mortality risk was greater for male patients in Group 4 (HR 1.79, 95% CI 1.20–2.67, *p* = 0.005).

### Sensitivity analysis

We chose the 3–0,0,0 model for further sensitivity analysis. This model had three steady-state trajectories including high, medium and low level, and guaranteed the sufficient sample size of the high-level trajectory group (Supplementary Figure S6). The results of sensitivity analysis were consistent with the primary analysis. KM survival curves revealed that the medium-level trajectory group had the lowest mortality rates within 365 days (Supplementary Figure S7). Multivariate Cox regression revealed that compared with the medium-level trajectory group, the low-level trajectory group exhibited increased 30-day (HR 1.36, 95% CI 1.10–1.69, *p* = 0.005), 90-day (HR 1.44, 95% CI 1.21–1.73, *p* < 0.001), 180-day (HR 1.43, 95% CI 1.21–1.70, *p* < 0.001), and 365-day (HR 1.43, 95% CI 1.22–1.68, *p* < 0.001) mortality risk (Supplementary Table S10). And the high-level group was associated with elevated 365-day mortality risk (HR 1.26, 95% CI 1.02–1.55, *p* = 0.033). Multivariate linear regression analysis showed that the low-level trajectory group was significantly correlated with prolonged hospital LOS (*β* 3.47, 95% CI 1.33–4.77, *p* < 0.001), but no significant association was observed between the high-level group and hospital LOS. Finally, the subgroup analysis confirmed the primary interactions, revealing that the association between the high-level trajectory group and short-term mortality significantly interacted with age and sex (Supplementary Tables S11–S14).

## Discussion

In the present study, we focused on early SA-AKI and determined the dynamic levels of serum albumin following the onset of early SA-AKI by the GBTM. We identified five interesting step-arranged trajectories, including four zero-slope trajectories (at levels of 2.0 g/dL, 2.5 g/dL, 3.0 g/dL, and 3.5 g/dL) and a slightly ascending trajectory (at a level of > 4.1 g/dL). This model is different from traditional trajectory model and closer to steady-state subgroup identification or baseline-stratified trajectory subgroups. Thus, in this study, we described the model as baseline-stratified trajectory subgroups. These results contribute to the study of the correlation between different baseline-stratified trajectory subgroups of serum albumin and prognosis of early SA-AKI. Most importantly, we observed some albumin trajectories significantly correlated with short- or/and long-term mortality risk in sepsis patients with early SA-AKI.

There is a consensus that hypoproteinemia is related to poor prognosis of various diseases (15, 23–26). Our study revealed a similar trend from the perspective of dynamic albumin levels, with albumin trajectories at levels of 2.0 g/dL and 2.5 g/dL associated with increased 90-, 180-, and 365-day mortality risk among sepsis patients with early SA-AKI. Moreover, the trajectory at a level of 2.0 g/dL was also significantly related to 30-day mortality rate. In most ICU settings, severe hypoalbuminemia typically triggers therapeutic interventions due to its association with worsened outcomes. In this study, patients with the albumin trajectory at a level of 2.0 g/dL received conservative albumin infusion and relatively positive nutritional support potentially due to higher median age and proportion of malignancy. Tie and colleagues reported that sepsis patients with persistently low serum albumin concentrations (<3.0 g/dL) had the highest mortality rate according to the trajectory of serum albumin concentration (13). Compared with their study, our research may be more helpful for the assessment of albumin risk thresholds because of our zero-slope trajectory gradients. Another retrospective study focused on sepsis patients with AKI who underwent RRT and reported a negative correlation between albumin levels and both 28-day and 90-day mortality (27). Cao et al. reported a significant association between decreased albumin levels and elevated mortality rates at 28, 60, 180, and 365 days in critically ill patients with sepsis when the serum albumin concentration was ≤2.6 g/dL (12). In this study, hospital LOS after AKI in patients with the albumin trajectory at a level of >4.1 g/dL was much more closed to that in patients with the albumin trajectories at levels of 2.0 g/dL and 2.5 g/dL. Multivariate linear regression analysis revealed the significant association between both the albumin trajectories at levels of 2.0 and >4.1 g/dL and prolonged hospital LOS. Regrettably, sensitivity analysis failed to confirm the association between high-level albumin trajectory and prolonged hospital LOS. Moreover, our results further revealed that patients with the albumin trajectory at a level of 2.0 g/dL or 2.5 g/dL was not associated with extended ICU LOS, increased days of RRT and prolonged durations of IMV. Limited evidence reveals adverse findings in both septic and non-septic patients (28, 29).

However, in this study, we also found that compared with patients with the trajectory at a level of 3.0 g/dL, early SA-AKI patients with the trajectory at a level of >4.1 g/dL exhibited higher 365-day mortality risk. Unfortunately, our research did not find any advantage of high serum albumin levels in terms of short-term or long-term survival. These findings seem to challenge the prevailing belief among clinicians that high serum albumin levels are correlated with more favorable prognoses. Our research cannot infer this causal relationship that high serum albumin levels lead to increased 365-day mortality risk. Notably, in this study, the proportion of cirrhosis was as high as 75.9% in patients with the trajectory at a level of >4.1 g/dL. Although the high serum albumin levels in these patients were probably related to more albumin infusion, we did not find that the association between the trajectory at a level of >4.1 g/dL and 365-day mortality risk interacted with cirrhosis in the subgroup analysis. Although the use of albumin is helpful for preventing and treating AKI associated with hepatorenal syndrome, many controversies still exist, such as the dosage, frequency and maintenance concentration of serum albumin (30). Hu et al. reported that only when the serum albumin concentration is between 2.5 and 3.0 g/dL can initiating albumin infusion significantly benefit sepsis patients with cirrhosis (31). In patients with decompensated cirrhosis, maintaining serum albumin concentration at 3.0 g/dL or higher can lead to more serious adverse events, especially pulmonary edema and fluid overload (32). There may be other confounding factors that affect this result. However, the further sensitivity analysis proved that our result was not an accidental statistical result. In fact, some studies support our view to some extent. Some studies have revealed that in both sepsis and non-cardiac surgery patients, the use of albumin may increase the risk of AKI (16, 33). High serum albumin levels are independently associated with AKI, even in children (34, 35). Of cause, the opposite view also exists. Jiang et al. reported the key effect of serum albumin in predicting mortality in patients with COPD (36). Whereas serum albumin levels of >4.0 g/dL was a favorable factor for predicting acute kidney disease in their research.

Although albumin is widely used in fluid resuscitation in sepsis, the optimal target interval for albumin levels is unclear. Although the baseline-stratified trajectories we found cannot be directly used as target levels of serum albumin, they can provide a direction for the follow-up research and can be used as a tool for risk stratification and prediction of short-term and long-term mortality. The comprehensive indexes developed on the basis of serum albumin are also outstanding in predicting poor prognosis. Lactate dehydrogenase-to-albumin ratio has been shown to be related to poor outcomes in patients with SA-AKI (37). In septic patients with malignancies, this indicator also demonstrates good ability to predict 28-day ICU mortality (38). Lactate-to-albumin ratio, another comprehensive indicator, is significantly associated with mortality risk in patients with SA-AKI (39). In addition, dynamics of oxidative stress and antioxidant capacity in sepsis patients correlate with renal outcomes (40). The combination of these oxidative stress and antioxidant markers and serum albumin may become another research direction in developing predictive models of SA-AKI.

Moreover, our subgroup analysis confirmed the correlation between the high-level trajectory groups and short-term mortality significantly interacted with age and sex. Patients under 65 years old had greater short-term mortality risk, which seems to contradict our understanding. Although advanced age is linked to a higher mortality rate among patients with sepsis, it is more strongly influenced by age-related comorbidities. Owing to the significant heterogeneity of sepsis, eliminating all the confounding factors in clinical research is difficult. Indeed, elderly patients with sepsis demonstrate reduced activation and dysfunction of endothelial cells and exhibit a sepsis-induced decrease in the expression of cytokine signaling pathways but heightened expression of genes associated with hemostasis (41). These mechanisms may lead to less organ injury in elderly patients than in younger patients during the acute stage of sepsis. This hypothesis needs to be verified by prospective research or animal experiments. As for sex difference, only one trajectory group in this study exhibited increased 30-day mortality risk in male patients. Peng et al. reported there was no difference in ICU or hospital mortality rate between male and female patients with SA-AKI (42). Different hormone levels caused by sex difference may have different effects on the outcomes of sepsis (43). Male patients appear to be associated with more adverse events and poor outcomes (44). However, this contention has remained a subject of ongoing debate.

This study focuses on the dynamic changes in serum albumin levels following the onset of early SA-AKI and provides new insight into risk stratification strategy in sepsis patients. Although this was a retrospective study, we further controlled the confounding factors by elucidating the temporal configuration of the research question. Certainly, this study has several limitations. First, we only included patients who had at least three records of serum albumin concentration in order to ensure that the trajectory model could more truly reflect the dynamic level of serum albumin. This inclusion criterion may enrich for specific patterns of illness severity, monitoring intensity and albumin administration, and exclude many early deaths. Whether our findings can be generalized to all patients with early SA-AKI needs to be verified by further prospective study. Second, each trajectory in this study represents the average level of serum albumin changing over time, which cannot be directly used as the target level for treatment. Third, despite our best efforts to adjust for confounding variables, the inherent complexity of critically ill patients means that there may still be residual and unmeasured confounding factors capable of influencing our results. Finally, this is a retrospective study from a single-center database in the United States. The details of institutional protocols for hypoalbuminemia management, including albumin infusion, nutritional support and associated timing of measurements, during the study period remains unclear and may affect the outcomes. Thus, whether our findings can be generalized to other settings necessitates external and multi-center validation, particularly within prospective cohorts, to guarantee general applicability.

## Conclusion

This study revealed that the baseline-stratified trajectory subgroups of serum albumin levels following early SA-AKI were significantly association with hospital LOS and short- and long-term mortality risk and could serve as a valuable indicator for guiding risk stratification and predicting prognosis in critically ill patients with early SA-AKI. Notably, clinicians need to maintain extra vigilance in the management of sepsis patients with the albumin trajectory at a level of >4.1 g/dL after SA-AKI onset.

## Data Availability

The datasets presented in this study can be found in online repositories. The names of the repository/repositories and accession number(s) can be found at: the data analyzed in this study are publicly available at: https://physionet.org/content/mimiciv/3.1/.

## Glossary

SA-AKI: sepsis-associated acute kidney injury
ICU: intensive care unit
MIMIC: Medical Information Mart for Intensive Care
GBTM: group-based trajectory modeling
KM: Kaplan–Meier
HR: hazard ratio
CI: confidence interval
KDIGO: Kidney Disease: Improving Global Outcomes
SOFA: Sequential Organ Failure Assessment
SQL: Structured Query Language
SAPS II: Simplified Acute Physiology Score II
CCI: Charlson comorbidity index
IMV: invasive mechanical ventilation
RRT: renal replacement therapy
LOS: length of stay
BIC: Bayesian Information Criterion
AIC: Akaike Information Criterion
AvePP: average posterior probability
OCC: odds of correct classification
IQR: interquartile range
SD: standard deviation
MICU: medical intensive care unit
SICU: surgical intensive care unit
MICU/SICU: medical/surgical intensive care unit
CVICU: cardiac vascular intensive care unit
CCU: coronary care unit
CKD: chronic kidney disease
COPD: chronic obstructive pulmonary disease
NMAP: non-invasive mean arterial pressure
SpO_2_: peripheral oxygen saturation
WBC: white blood cell count
ALT: alanine aminotransferase
AST: aspartate aminotransferase
PT: prothrombin time
PTT: partial thromboplastin time
