# Supplementary material for: Association between baseline-stratified trajectory subgroups of serum albumin and outcomes in critically ill patients with early sepsis-associated acute kidney injury: a retrospective study
Source: Front Med (Lausanne). 2026 May 29;13:1722950. doi: 10.3389/fmed.2026.1722950 (PMC13259871; doi:10.3389/fmed.2026.1722950)
Supplement: Supplementary file 1 [file Data_Sheet_1.pdf]

# Supplementary Materials

**Table S1.** Percentage of missing data in the variables of interest.

| Variables | Percentage (n=1914) |
|---|---|
| Age, years | 0% |
| Sex | 0% |
| Race | 0% |
| Heart rate, bpm | 0% |
| Respiratory rate, bpm | 0% |
| NMAP, mmHg | 0.84% |
| $SpO_2$, % | 0.05% |
| WBC, $\times 10^9$/L | 0.52% |
| Hemoglobin, g/L | 0.52% |
| Platelet count, $\times 10^9$/L | 0.47% |
| Total bilirubin, mg/dL | 1.78% |
| ALT, IU/L | 2.19% |
| AST, IU/L | 1.31% |
| Creatinine, mg/dL | 0.05% |
| Urea nitrogen, mg/dL | 0% |
| PT, s | 1.78% |
| PTT, s | 2.04% |
| Sodium, mEq/L | 0% |
| Potassium, mEq/L | 0.10% |
| Chloride, mEq/L | 0% |
| Total calcium, mg/dL | 0.10% |
| Glucose, mg/dL | 0% |
| Anion gap, mEq/L | 0.05% |
| pH | 3.76% |
| PCO2, mmHg | 5.02% |
| PO2, mmHg | 5.02% |
| Lactate, mmol/L | 4.65% |
| Fluid balance, mL | 23.62 |
| SOFA score | 0% |
| SAPS II | 0% |
| CCI score | 0% |
| ICU LOS after AKI, d | 0% |
| Hospital LOS after AKI, d | 0% |

Abbreviations: NMAP, non-invasive mean arterial pressure; $SpO_2$, peripheral oxygen saturation; WBC, white blood cell count; ALT, alanine aminotransferase; AST, aspartate aminotransferase; PT, prothrombin time; PTT, partial thromboplastin time; SOFA, Sequential Organ Failure Assessment; SAPS, simplified acute physiology score; CCI, Charlson comorbidity index; LOS, length of stay; AKI, acute kidney

injury.

**Table S2.** Results of trajectory model parameter estimation with statistical significance.

| Models | Parameter | Estimate | SE | t | *p* |
|---|---|---|---|---|---|
| 2~0,0 | | | | | |
| Group 1 | Intercept | 2.43971 | 0.00958 | 254.790 | 0.0000 |
| Group 2 | Intercept | 3.32409 | 0.01243 | 267.382 | 0.0000 |
| 3~0,0,0 | | | | | |
| Group 1 | Intercept | 2.21462 | 0.01154 | 191.874 | 0.0000 |
| Group 2 | Intercept | 2.89566 | 0.01109 | 261.217 | 0.0000 |
| Group 3 | Intercept | 3.68700 | 0.01473 | 250.276 | 0.0000 |
| 4~0,0,0,0 | | | | | |
| Group 1 | Intercept | 2.05632 | 0.01365 | 150.605 | 0.0000 |
| Group 2 | Intercept | 2.65002 | 0.01394 | 190.163 | 0.0000 |
| Group 3 | Intercept | 3.21316 | 0.01591 | 201.933 | 0.0000 |
| Group 4 | Intercept | 3.96450 | 0.02147 | 184.694 | 0.0000 |
| 5~0,0,0,0,1 | | | | | |
| Group 1 | Intercept | 1.97343 | 0.01493 | 132.143 | 0.0000 |
| Group 2 | Intercept | 2.50306 | 0.01390 | 180.130 | 0.0000 |
| Group 3 | Intercept | 2.98227 | 0.01296 | 230.060 | 0.0000 |
| Group 4 | Intercept | 3.52174 | 0.01570 | 224.341 | 0.0000 |
| Group 5 | Intercept | 4.12938 | 0.04434 | 93.130 | 0.0000 |
| | Linear | 0.03116 | 0.01022 | 3.049 | 0.0023 |
| 6~0,0,0,0,0,1 | | | | | |
| Group 1 | Intercept | 1.94214 | 0.01484 | 130.833 | 0.0000 |
| Group 2 | Intercept | 2.44800 | 0.01437 | 170.397 | 0.0000 |
| Group 3 | Intercept | 2.89357 | 0.01586 | 182.420 | 0.0000 |
| Group 4 | Intercept | 3.34809 | 0.02017 | 165.973 | 0.0000 |
| Group 5 | Intercept | 3.86996 | 0.02221 | 174.276 | 0.0000 |
| Group 6 | Intercept | 4.25018 | 0.07304 | 58.189 | 0.0000 |
| | Linear | 0.08800 | 0.01620 | 5.433 | 0.0000 |

Abbreviations: SE, standard error.

**Table S3.** Performance of different trajectory models.

| trajectory models | BIC | AIC | OCC | AvePP per group | Proportion per group (%) |
|---|---|---|---|---|---|
| 2~0,0 | -5704.53 | -5701.64 | 12.6/20.1 | 0.95/0.93 | 60.2/39.8 |
| 3~0,0,0 | -4751.71 | -4747.37 | 26.5/12.6/87.4 | 0.93/0.93/0.94 | 33.4/51.4/15.2 |
| 4~0,0,0,0 | -4297.76 | -4291.99 | 46.3/13.1/21.2/218.1 | 0.92/0.91/0.90/0.94 | 19.9/43.6/29.8/6.7 |
| 5~0,0,0,0,1 | -4023.96 | -4016.02 | 78.2/14.4/15.1/93.9/543.9 | 0.93/0.88/0.89/0.94/0.94 | 14.5/33.7/34.8/14.3/2.8 |
| 6~0,0,0,0,0,1 | -3921.60 | -3912.22 | 79.1/17.1/13.1/32.4/151.7/1902.1 | 0.92/0.88/0.87/0.87/0.90/0.95 | 12.7/29.9/33.8/17.1/5.6/0.9 |

Abbreviations: BIC, Bayesian information criterion; AIC, Akaike Information criterion; OCC, odds of correct classification; AvePP, average posterior probability.

**Table S4.** Results of Kruskal-Wallis H-test after multiple interpolation between the 5 trajectory groups.

| Variables | Raw data | MI 1 | MI 2 | MI 3 | MI 4 | M5 |
|---|---|---|---|---|---|---|
| NMAP, mmHg | <.001 | 0.002 | <.001 | <.001 | <.001 | <.001 |
| $SpO_2$, % | 0.006 | 0.007 | 0.007 | 0.006 | 0.007 | 0.007 |
| WBC, $10^9$/L | <.001 | <.001 | <.001 | <.001 | <.001 | <.001 |
| Platelet, $10^9$/L | <.001 | <.001 | <.001 | <.001 | <.001 | <.001 |
| Hemoglobin, g/dL | 0.013 | 0.018 | 0.016 | 0.009 | 0.007 | 0.009 |
| Total bilirubin, mg/dL | <.001 | <.001 | <.001 | <.001 | <.001 | <.001 |
| ALT, IU/L | 0.002 | 0.004 | 0.004 | 0.004 | 0.003 | 0.004 |
| AST, IU/L | 0.002 | 0.003 | 0.004 | 0.003 | 0.003 | 0.004 |
| Creatinine, mg/dL | 0.029 | 0.029 | 0.028 | 0.028 | 0.027 | 0.028 |
| PT, s | <.001 | <.001 | <.001 | <.001 | <.001 | <.001 |
| PTT, s | 0.004 | 0.007 | 0.003 | 0.005 | 0.008 | 0.004 |
| Potassium, mEq/L | 0.110 | 0.112 | 0.112 | 0.104 | 0.109 | 0.114 |
| Total calcium, mg/dL | <.001 | <.001 | <.001 | <.001 | <.001 | <.001 |
| Anion gap, mEq/L | 0.058 | 0.057 | 0.059 | 0.058 | 0.058 | 0.059 |
| pH | <.001 | <.001 | <.001 | <.001 | <.001 | <.001 |
| $PCO_2$, mmHg | <.001 | <.001 | 0.002 | <.001 | 0.001 | <.001 |
| $PO_2$, mmHg | 0.404 | 0.408 | 0.158 | 0.161 | 0.477 | 0.368 |
| Lactate, mmol/L | 0.389 | 0.567 | 0.645 | 0.489 | 0471 | 0.602 |

Abbreviations: MI, multiple interpolation; NMAP, non-invasive mean arterial pressure; $SpO_2$, peripheral oxygen saturation; WBC, white blood cell count; ALT, alanine aminotransferase; AST, aspartate aminotransferase.

**Table S5.** Linear regression analysis for the association between trajectory groups and ICU LOS, hospital LOS, RRT days and duration of IMV after early SA-AKI.

| Outcomes | Unadjusted | | Adjusted | |
|---|---|---|---|---|
| | β (95%CI) | *p* | β (95%CI) | *p* |
| ICU LOS | | | | |
| Group 1 | 2.34 (1.00~3.69) | <.001 | 0.92 (-0.29~2.12) | 0.135 |
| Group 2 | 1.60 (0.58~2.62) | 0.002 | 0.88 (0.00 ~1.77) | 0.050 |
| Group 3 | 0.00 (Reference) | | 0.00 (Reference) | |
| Group 4 | -0.18 (-1.55~1.19) | 0.800 | -0.01 (-1.18~1.12) | 0.985 |
| Group 5 | 0.48 (-2.16~3.11) | 0.723 | 0.30 (-1.08~3.52) | 0.299 |
| Hospital LOS | | | | |
| Group 1 | 4.95 (3.65~8.44) | <.001 | 3.36 (1.76~6.70) | <.001 |
| Group 2 | 2.60 (0.59~4.22) | 0.009 | 1.67 (-0.27~3.35) | 0.096 |
| Group 3 | 0.00 (Reference) | | 0.00 (Reference) | |
| Group 4 | 0.20 (-2.19~2.68) | 0.843 | 0.22 (-2.13~2.68) | 0.824 |
| Group 5 | 3.45 (3.56~12.94) | <.001 | 3.47 (3.64~13.09) | <.001 |
| RRT days | | | | |
| Group 1 | 0.79 (0.09~1.48) | 0.027 | 0.26 (-0.38~0.90) | 0.425 |
| Group 2 | 0.66 (0.13~1.18) | 0.014 | 0.35 (-0.12~0.82) | 0.149 |
| Group 3 | 0.00 (Reference) | | 0.00 (Reference) | |
| Group 4 | 0.20 (-0.51~0.91) | 0.581 | -0.04 (-0.66~0.59) | 0.907 |
| Group 5 | 1.03 (-0.33~2.39) | 0.139 | 0.52 (-0.70~1.74) | 0.407 |
| Duration of IMV | | | | |
| Group 1 | 39.98 (15.86~64.10) | 0.001 | 10.01 (-12.62~32.63) | 0.386 |
| Group 2 | 25.55 (7.26~43.84) | 0.006 | 8.53 (-8.10~25.17) | 0.315 |
| Group 3 | 0.00 (Reference) | | 0.00 (Reference) | |
| Group 4 | -1.87 (-26.43~22.69) | 0.881 | 0.76 (-21.30~22.82) | 0.946 |
| Group 5 | 2.10 (-45.17~49.38) | 0.931 | 7.91 (-35.31~51.14) | 0.720 |

Abbreviations: ICU, intensive care unit; LOS, length of stay; RRT, renal replacement therapy; IMV, invasive mechanical ventilation; SA-AKI, sepsis-associated acute kidney injury; CI, confidence interval. The covariates used for adjustment were as follows: age, sex, ICU type, SAPS II, cirrhosis, malignancy, COPD, acute pancreatitis, heart rate, NMAP, respiratory rate, $SpO_2$, WBC, platelet, hemoglobin, total bilirubin, ALT, AST, urea nitrogen, creatinine, PT, PTT, sodium, total calcium, chloride, glucose, anion gap, pH, $PCO_2$, albumin infusion, norepinephrine, phenylephrine, vasopressin, enteral nutrition, parenteral nutrition, IMV after AKI.

**Table S6.** Subgroup analysis for the association between trajectory groups and 30-day mortality.

| Trajectory | Subgroup | HR (95%CI) | *p* | P for interaction |
|---|---|---|---|---|
| | **Age** | | | |
| Group 1 | <65 years | 1.73 (1.05~2.85) | 0.032 | 0.792 |
| | ≥65 years | 1.86 (1.28~2.69) | 0.001 | |
| Group 2 | <65 years | 0.98 (0.65~1.57) | 0.900 | 0.482 |
| | ≥65 years | 1.33 (0.97~1.81) | 0.077 | |
| Group 3 | <65 years | 1.00 (Reference) | | |
| | ≥65 years | 1.00 (Reference) | | |
| Group 4 | <65 years | 1.61 (1.05~2.47) | 0.031 | 0.005 |
| | ≥65 years | 0.59 (0.34~1.02) | 0.059 | |
| Group 5 | <65 years | 1.92 (0.99~3.72) | 0.054 | 0.033 |
| | ≥65 years | 0.46 (0.17~1.27) | 0.133 | |
| | **Sex** | | | |
| Group 1 | Male | 2.15 (1.45~3.17) | <.001 | 0.056 |
| | Female | 1.82 (1.14~2.92) | 0.013 | |
| Group 2 | Male | 1.25 (0.90~1.75) | 0.188 | 0.577 |
| | Female | 1.27 (0.88~1.84) | 0.204 | |
| Group 3 | Male | 1.00 (Reference) | | |
| | Female | 1.00 (Reference) | | |
| Group 4 | Male | 1.79 (1.20~2.67) | 0.005 | 0.004 |
| | Female | 0.63 (0.35~1.12) | 0.117 | |
| Group 5 | Male | 1.15 (0.56~2.39) | 0.707 | 0.978 |
| | Female | 0.85 (0.35~2.03) | 0.710 | |
| | **Pneumonia** | | | |
| Group 1 | No | 2.26 (1.47~3.48) | <.001 | 0.243 |
| | Yes | 1.38 (0.91~2.11) | 0.131 | |
| Group 2 | No | 1.39 (0.97~1.99) | 0.075 | 0.346 |
| | Yes | 1.00 (0.71~1.41) | 0.985 | |
| Group 3 | No | 1.00 (Reference) | | |
| | Yes | 1.00 (Reference) | | |
| Group 4 | No | 1.48 (0.94~2.34) | 0.091 | 0.108 |
| | Yes | 0.92 (0.57~1.48) | 0.721 | |
| Group 5 | No | 1.11 (0.52~2.37) | 0.784 | 0.446 |
| | Yes | 1.19 (0.53~2.66) | 0.680 | |
| | **Hypertension** | | | |
| Group 1 | No | 1.90 (1.31~2.74) | <.001 | 0.735 |
| | Yes | 2.34 (1.37~4.00) | 0.002 | |
| Group 2 | No | 1.30 (0.97~1.75) | 0.082 | 0.282 |
| | Yes | 1.03 (0.66~1.61) | 0.882 | |
| Group 3 | No | 1.00 (Reference) | | |

| | | | | |
|---|---|---|---|---|
| | Yes | 1.00 (Reference) | | |
| Group 4 | No | 1.12 (0.80~1.75) | 0.412 | 0.981 |
| | Yes | 1.18 (0.64~2.18) | 0.599 | |
| Group 5 | No | 1.12 (0.57~2.20) | 0.740 | 0.616 |
| | Yes | 0.92 (0.33~2.59) | 0.875 | |
| | **Diabetes** | | | |
| Group 1 | No | 2.02 (1.40~2.92) | <.001 | 0.357 |
| | Yes | 1.95 (1.16~3.30) | 0.012 | |
| Group 2 | No | 1.23 (0.91~1.66) | 0.182 | 0.605 |
| | Yes | 1.14 (0.74~1.77) | 0.549 | |
| Group 3 | No | 1.00 (Reference) | | |
| | Yes | 1.00 (Reference) | | |
| Group 4 | No | 1.24 (0.84~1.84) | 0.283 | 0.650 |
| | Yes | 1.05 (0.58~1.89) | 0.879 | |
| Group 5 | No | 1.26 (0.66~2.39) | 0.490 | 0.738 |
| | Yes | 1.54 (0.54~4.34) | 0.417 | |
| | **Cirrhosis** | | | |
| Group 1 | No | 2.29 (1.61~3.24) | <.001 | 0.322 |
| | Yes | 1.25 (0.67~2.35) | 0.488 | |
| Group 2 | No | 1.34 (0.99~1.81) | 0.060 | 0.661 |
| | Yes | 0.98 (0.63~1.52) | 0.917 | |
| Group 3 | No | 1.00 (Reference) | | |
| | Yes | 1.00 (Reference) | | |
| Group 4 | No | 0.65 (0.36~1.19) | 0.164 | 0.059 |
| | Yes | 1.38 (0.89~2.14) | 0.146 | |
| Group 5 | No | 0.35 (0.05~2.64) | 0.309 | 0.236 |
| | Yes | 1.35 (0.74~2.49) | 0.331 | |

Abbreviations: HR, hazard ratio; CI, confidence interval. Subgroup analysis was adjusted for age, sex, ICU type, SAPS II, cirrhosis, malignancy, COPD, acute pancreatitis, heart rate, NMAP, respiratory rate, $SpO_2$, WBC, platelet, hemoglobin, total bilirubin, ALT, AST, urea nitrogen, creatinine, PT, PTT, sodium, total calcium, chloride, glucose, anion gap, pH, $PCO_2$, albumin infusion, norepinephrine, phenylephrine, vasopressin, enteral nutrition, parenteral nutrition, IMV after AKI.

**Table S7.** Subgroup analysis for the association between trajectory groups and 90-day mortality.

| Trajectory | Subgroup | HR (95%CI) | *p* | P for interaction |
|---|---|---|---|---|
| | **Age** | | | |
| Group 1 | <65 years | 1.97 (1.31~2.97) | 0.001 | 0.720 |
| | ≥65 years | 1.96 (1.44~2.67) | <.001 | |
| Group 2 | <65 years | 1.16 (0.84~1.61) | 0.367 | 0.971 |
| | ≥65 years | 1.32 (1.01~1.71) | 0.039 | |
| Group 3 | <65 years | 1.00 (Reference) | | |
| | ≥65 years | 1.00 (Reference) | | |
| Group 4 | <65 years | 1.45 (0.99~2.11) | 0.056 | 0.029 |
| | ≥65 years | 0.72 (0.47~1.11) | 0.135 | |
| Group 5 | <65 years | 2.12 (1.22~3.67) | 0.008 | 0.037 |
| | ≥65 years | 0.75 (0.36~1.54) | 0.427 | |
| | **Sex** | | | |
| Group 1 | Male | 1.93 (1.40~2.67) | <.001 | 0.654 |
| | Female | 2.36 (1.59~3.49) | <.001 | |
| Group 2 | Male | 1.16 (0.88~1.52) | 0.285 | 0.385 |
| | Female | 1.52 (1.11~2.10) | 0.010 | |
| Group 3 | Male | 1.00 (Reference) | | |
| | Female | 1.00 (Reference) | | |
| Group 4 | Male | 1.31 (0.93~1.86) | 0.128 | 0.243 |
| | Female | 0.90 (0.57~1.43) | 0.647 | |
| Group 5 | Male | 1.30 (0.74~2.27) | 0.367 | 0.525 |
| | Female | 1.28 (0.63~2.62) | 0.495 | |
| | **Pneumonia** | | | |
| Group 1 | No | 2.07 (1.46~2.94) | <.001 | 0.545 |
| | Yes | 1.61 (1.13~2.30) | 0.009 | |
| Group 2 | No | 1.35 (1.01~1.80) | 0.044 | 0.397 |
| | Yes | 1.06 (0.80~1.42) | 0.686 | |
| Group 3 | No | 1.00 (Reference) | | |
| | Yes | 1.00 (Reference) | | |
| Group 4 | No | 1.24 (0.84~1.84) | 0.279 | 0.531 |
| | Yes | 1.02 (0.69~1.52) | 0.921 | |
| Group 5 | No | 1.35 (0.75~2.45) | 0.321 | 0.522 |
| | Yes | 1.57 (0.83~2.97) | 0.164 | |
| | **Hypertension** | | | |
| Group 1 | No | 2.16 (1.60~2.92) | <.001 | 0.885 |
| | Yes | 1.93 (1.23~3.03) | 0.005 | |
| Group 2 | No | 1.39 (1.09~1.78) | 0.008 | 0.173 |
| | Yes | 0.98 (0.67~1.44) | 0.930 | |
| Group 3 | No | 1.00 (Reference) | | |

| | | | | |
|---|---|---|---|---|
| | Yes | 1.00 (Reference) | | |
| Group 4 | No | 1.10 (0.79~1.53) | 0.570 | 0.745 |
| | Yes | 1.16 (0.68~1.98) | 0.596 | |
| Group 5 | No | 1.23 (0.72~2.12) | 0.449 | 0.954 |
| | Yes | 1.70 (0.79~3.68) | 0.175 | |
| | **Diabetes** | | | |
| Group 1 | No | 2.01 (1.46~2.75) | <.001 | 0.660 |
| | Yes | 2.06 (1.37~3.10) | <.001 | |
| Group 2 | No | 1.35 (1.04~1.74) | 0.022 | 0.271 |
| | Yes | 1.09 (0.76~1.55) | 0.653 | |
| Group 3 | No | 1.00 (Reference) | | |
| | Yes | 1.00 (Reference) | | |
| Group 4 | No | 1.18 (0.84~1.67) | 0.345 | 0.678 |
| | Yes | 0.93 (0.58~1.50) | 0.774 | |
| Group 5 | No | 1.70 (1.04~2.80) | 0.035 | 0.847 |
| | Yes | 1.48 (0.62~3.55) | 0.381 | |
| | **Cirrhosis** | | | |
| Group 1 | No | 2.48 (1.85~3.32) | <.001 | 0.111 |
| | Yes | 1.29 (0.74~2.22) | 0.368 | |
| Group 2 | No | 1.43 (1.11~1.84) | 0.006 | 0.465 |
| | Yes | 1.06 (0.74~1.53) | 0.747 | |
| Group 3 | No | 1.00 (Reference) | | |
| | Yes | 1.00 (Reference) | | |
| Group 4 | No | 0.67 (0.41~1.10) | 0.114 | 0.042 |
| | Yes | 1.35 (0.93~1.96) | 0.118 | |
| Group 5 | No | 1.03 (0.36~2.94) | 0.957 | 0.561 |
| | Yes | 1.45 (0.88~2.38) | 0.150 | |

Abbreviations: HR, hazard ratio; CI, confidence interval. Subgroup analysis was adjusted for age, sex, ICU type, SAPS II, cirrhosis, malignancy, COPD, acute pancreatitis, heart rate, NMAP, respiratory rate, $SpO_2$, WBC, platelet, hemoglobin, total bilirubin, ALT, AST, urea nitrogen, creatinine, PT, PTT, sodium, total calcium, chloride, glucose, anion gap, pH, $PCO_2$, albumin infusion, norepinephrine, phenylephrine, vasopressin, enteral nutrition, parenteral nutrition, IMV after AKI.

**Table S8.** Subgroup analysis for the association between trajectory groups and 180-day mortality.

| Trajectory | Subgroup | HR (95%CI) | *p* | P for interaction |
|---|---|---|---|---|
| | **Age** | | | |
| Group 1 | <65 years | 2.02 (1.37~2.98) | <.001 | 0.593 |
| | ≥65 years | 1.86 (1.39~2.49) | <.001 | |
| Group 2 | <65 years | 1.19 (0.87~1.61) | 0.272 | 0.857 |
| | ≥65 years | 1.28 (1.00~1.63) | 0.049 | |
| Group 3 | <65 years | 1.00 (Reference) | | |
| | ≥65 years | 1.00 (Reference) | | |
| Group 4 | <65 years | 1.32 (0.92~1.89) | 0.138 | 0.074 |
| | ≥65 years | 0.81 (0.55~1.18) | 0.270 | |
| Group 5 | <65 years | 1.89 (1.10~3.27) | 0.022 | 0.061 |
| | ≥65 years | 0.84 (0.43~1.63) | 0.602 | |
| | **Sex** | | | |
| Group 1 | Male | 1.90 (1.40~2.57) | <.001 | 0.689 |
| | Female | 2.23 (1.53~3.32) | <.001 | |
| Group 2 | Male | 1.12 (0.87~1.45) | 0.374 | 0.213 |
| | Female | 1.53 (1.13~2.06) | 0.006 | |
| Group 3 | Male | 1.00 (Reference) | | |
| | Female | 1.00 (Reference) | | |
| Group 4 | Male | 1.28 (0.92~1.77) | 0.139 | 0.245 |
| | Female | 0.91 (0.59~1.40) | 0.665 | |
| Group 5 | Male | 1.22 (0.72~2.09) | 0.457 | 0.558 |
| | Female | 1.28 (0.63~2.58) | 0.495 | |
| | **Pneumonia** | | | |
| Group 1 | No | 1.97 (1.42~2.74) | <.001 | 0.575 |
| | Yes | 1.62 (1.15~2.28) | 0.006 | |
| Group 2 | No | 1.36 (1.04~1.78) | 0.026 | 0.296 |
| | Yes | 1.05 (0.80~1.38) | 0.736 | |
| Group 3 | No | 1.00 (Reference) | | |
| | Yes | 1.00 (Reference) | | |
| Group 4 | No | 1.25 (0.87~1.79) | 0.233 | 0.461 |
| | Yes | 1.01 (0.70~1.48) | 0.940 | |
| Group 5 | No | 1.40 (0.80~2.45) | 0.240 | 0.415 |
| | Yes | 1.40 (0.74~2.63) | 0.298 | |
| | **Hypertension** | | | |
| Group 1 | No | 2.03 (1.54~2.70) | <.001 | 0.997 |
| | Yes | 1.94 (1.26~3.01) | 0.003 | |
| Group 2 | No | 1.31 (1.05~1.65) | 0.019 | 0.402 |
| | Yes | 1.08 (0.75~1.54) | 0.690 | |
| Group 3 | No | 1.00 (Reference) | | |

| | | | | |
|---|---|---|---|---|
| | Yes | 1.00 (Reference) | | |
| Group 4 | No | 1.12 (0.83~1.51) | 0.467 | 0.587 |
| | Yes | 1.08 (0.64~1.83) | 0.767 | |
| Group 5 | No | 1.24 (0.74~2.08) | 0.415 | 0.872 |
| | Yes | 1.60 (0.75~3.41) | 0.222 | |
| | **Diabetes** | | | |
| Group 1 | No | 1.88 (1.39~2.54) | <.001 | 0.630 |
| | Yes | 1.98 (1.35~2.89) | <.001 | |
| Group 2 | No | 1.30 (1.03~1.66) | 0.030 | 0.206 |
| | Yes | 1.07 (0.77~1.49) | 0.676 | |
| Group 3 | No | 1.00 (Reference) | | |
| | Yes | 1.00 (Reference) | | |
| Group 4 | No | 1.14 (0.82~1.58) | 0.450 | 0.941 |
| | Yes | 0.96 (0.63~1.48) | 0.864 | |
| Group 5 | No | 1.70 (1.06~2.74) | 0.029 | 0.610 |
| | Yes | 1.23 (0.52~2.91) | 0.645 | |
| | **Cirrhosis** | | | |
| Group 1 | No | 2.30 (1.74~3.02) | <.001 | 0.256 |
| | Yes | 1.72 (1.07~2.76) | 0.025 | |
| Group 2 | No | 1.37 (1.08~1.73) | 0.009 | 0.644 |
| | Yes | 1.10 (0.78~1.56) | 0.577 | |
| Group 3 | No | 1.00 (Reference) | | |
| | Yes | 1.00 (Reference) | | |
| Group 4 | No | 0.77 (0.50~1.18) | 0.224 | 0.088 |
| | Yes | 1.40 (0.84~2.33) | 0.194 | |
| Group 5 | No | 0.96 (0.34~2.73) | 0.942 | 0.470 |
| | Yes | 1.26 (0.88~1.80) | 0.203 | |

Abbreviations: HR, hazard ratio; CI, confidence interval. Subgroup analysis was adjusted for age, sex, ICU type, SAPS II, cirrhosis, malignancy, COPD, acute pancreatitis, heart rate, NMAP, respiratory rate, $SpO_2$, WBC, platelet, hemoglobin, total bilirubin, ALT, AST, urea nitrogen, creatinine, PT, PTT, sodium, total calcium, chloride, glucose, anion gap, pH, $PCO_2$, albumin infusion, norepinephrine, phenylephrine, vasopressin, enteral nutrition, parenteral nutrition, IMV after AKI.

**Table S9.** Subgroup analysis for the association between trajectory groups and 365-day mortality.

| Trajectory | Subgroup | HR (95%CI) | *p* | P for interaction |
|---|---|---|---|---|
| | **Age** | | | |
| Group 1 | <65 years | 1.99 (1.38~2.88) | <.001 | 0.754 |
| | ≥65 years | 1.77 (1.34~2.34) | <.001 | |
| Group 2 | <65 years | 1.20 (0.91~1.60) | 0.204 | 0.762 |
| | ≥65 years | 1.24 (0.98~1.56) | 0.073 | |
| Group 3 | <65 years | 1.00 (Reference) | | |
| | ≥65 years | 1.00 (Reference) | | |
| Group 4 | <65 years | 1.32 (0.95~1.85) | 0.103 | 0.111 |
| | ≥65 years | 0.92 (0.65~1.31) | 0.660 | |
| Group 5 | <65 years | 1.81 (1.08~3.01) | 0.024 | 0.059 |
| | ≥65 years | 0.95 (0.51~1.77) | 0.876 | |
| | **Sex** | | | |
| Group 1 | Male | 1.85 (1.39~2.47) | <.001 | 0.640 |
| | Female | 2.13 (1.49~3.03) | <.001 | |
| Group 2 | Male | 1.13 (0.89~1.43) | 0.319 | 0.268 |
| | Female | 1.45 (1.10~1.93) | 0.009 | |
| Group 3 | Male | 1.00 (Reference) | | |
| | Female | 1.00 (Reference) | | |
| Group 4 | Male | 1.41 (1.05~1.90) | 0.023 | 0.067 |
| | Female | 0.88 (0.59~1.33) | 0.557 | |
| Group 5 | Male | 1.35 (0.83~2.20) | 0.233 | 0.685 |
| | Female | 1.20 (0.61~2.35) | 0.593 | |
| | **Pneumonia** | | | |
| Group 1 | No | 1.80 (1.32~2.45) | <.001 | 0.983 |
| | Yes | 1.61 (1.18~2.28) | <.001 | |
| Group 2 | No | 1.28 (1.00~1.65) | 0.050 | 0.437 |
| | Yes | 1.06 (0.81~1.38) | 0.680 | |
| Group 3 | No | 1.00 (Reference) | | |
| | Yes | 1.00 (Reference) | | |
| Group 4 | No | 1.37 (0.99~1.88) | 0.055 | 0.181 |
| | Yes | 0.99 (0.68~1.42) | 0.939 | |
| Group 5 | No | 1.50 (0.91~2.50) | 0.115 | 0.304 |
| | Yes | 1.44 (0.79~2.66) | 0.238 | |
| | **Hypertension** | | | |
| Group 1 | No | 1.87 (1.43~2.44) | <.001 | 0.588 |
| | Yes | 2.05 (1.35~3.11) | <.001 | |
| Group 2 | No | 1.23 (0.99~1.52) | 0.058 | 0.911 |
| | Yes | 1.18 (0.84~1.66) | 0.335 | |
| Group 3 | | 1.00 (Reference) | | |

| | | | | |
|---|---|---|---|---|
| | | 1.00 (Reference) | | |
| Group 4 | No | 1.13 (0.85~1.50) | 0.393 | 0.941 |
| | Yes | 1.27 (0.79~2.04) | 0.322 | |
| Group 5 | No | 1.21 (0.75~1.97) | 0.438 | 0.445 |
| | Yes | 1.97 (0.99~3.92) | 0.055 | |
| | **Diabetes** | | | |
| Group 1 | No | 1.81 (1.36~2.41) | <.001 | 0.811 |
| | Yes | 1.90 (1.32~2.73) | <.001 | |
| Group 2 | No | 1.32 (1.05~1.65) | 0.017 | 0.115 |
| | Yes | 0.99 (0.72~1.36) | 0.953 | |
| Group 3 | No | 1.00 (Reference) | | |
| | Yes | 1.00 (Reference) | | |
| Group 4 | No | 1.17 (0.86~1.58) | 0.321 | 0.860 |
| | Yes | 1.05 (0.71~1.56) | 0.816 | |
| Group 5 | No | 1.74 (1.11~2.73) | 0.015 | 0.897 |
| | Yes | 1.40 (0.64~3.04) | 0.403 | |
| | **Cirrhosis** | | | |
| Group 1 | No | 2.19 (1.68~2.85) | <.001 | 0.254 |
| | Yes | 1.39 (0.86~2.26) | 0.180 | |
| Group 2 | No | 1.34 (1.07~1.67) | 0.011 | 0.561 |
| | Yes | 1.07 (0.78~1.49) | 0.666 | |
| Group 3 | No | 1.00 (Reference) | | |
| | Yes | 1.00 (Reference) | | |
| Group 4 | No | 0.90 (0.61~1.31) | 0.568 | 0.173 |
| | Yes | 1.29 (0.93~1.80) | 0.133 | |
| Group 5 | No | 1.36 (0.57~3.21) | 0.488 | 0.882 |
| | Yes | 1.39 (0.88~2.19) | 0.158 | |

Abbreviations: HR, hazard ratio; CI, confidence interval. Subgroup analysis was adjusted for age, sex, ICU type, SAPS II, cirrhosis, malignancy, COPD, acute pancreatitis, heart rate, NMAP, respiratory rate, $SpO_2$, WBC, platelet, hemoglobin, total bilirubin, ALT, AST, urea nitrogen, creatinine, PT, PTT, sodium, total calcium, chloride, glucose, anion gap, pH, $PCO_2$, albumin infusion, norepinephrine, phenylephrine, vasopressin, enteral nutrition, parenteral nutrition, IMV after AKI.

**Table S10.** Cox and linear regression analysis for the association between trajectory groups and outcomes in sensitivity analysis.

| Outcomes | Unadjusted | | Adjusted | |
|---|---|---|---|---|
| Mortalities | HR (95% CI) | *p* | HR (95% CI) | *p* |
| 30-day mortality | | | | |
| Group 1 | 1.50 (1.23~1.82) | <.001 | 1.36 (1.10~1.69) | 0.005 |
| Group 2 | 1.00 (Reference) | | 1.00 (Reference) | |
| Group 3 | 1.24 (0.94~1.62) | 0.123 | 1.15 (0.86~1.53) | 0.356 |
| 90-day mortality | | | | |
| Group 1 | 1.57 (1.33~1.85) | <.001 | 1.44 (1.21~1.73) | <.001 |
| Group 2 | 1.00 (Reference) | | 1.00 (Reference) | |
| Group 3 | 1.23 (0.98~1.54) | 0.072 | 1.17 (0.92~1.49) | 0.208 |
| 180-day mortality | | | | |
| Group 1 | 1.53 (1.31~1.79) | <.001 | 1.43 (1.21~1.70) | <.001 |
| Group 2 | 1.00 (Reference) | | 1.00 (Reference) | |
| Group 3 | 1.22 (0.99~1.51) | 0.063 | 1.17 (0.93~1.46) | 0.180 |
| 365-day mortality | | | | |
| Group 1 | 1.52 (1.31~1.76) | <.001 | 1.43 (1.22~1.68) | <.001 |
| Group 2 | 1.00 (Reference) | | 1.00 (Reference) | |
| Group 3 | 1.31 (1.07~1.59) | 0.008 | 1.26 (1.02~1.55) | 0.033 |
| Hospital LOS | β (95%CI) | *p* | β (95%CI) | *p* |
| Group 1 | 5.03 (2.65~6.04) | <.001 | 3.47 (1.33~4.77) | <.001 |
| Group 2 | 1.00 (Reference) | | 1.00 (Reference) | |
| Group 3 | 1.14 (-0.93~3.51) | 0.255 | 1.02 (-1.07~3.38) | 0.307 |

Abbreviations: HR, hazard ratio; CI, confidence interval; LOS, length of stay. The covariates used for adjustment were as follows: age, ICU type, SAPS II, cirrhosis, pneumonia, malignancy, COPD, MI, acute pancreatitis, heart rate, respiratory rate, $SpO_2$, WBC, platelet, hemoglobin, total bilirubin, ALT, AST, urea nitrogen, PT, PTT, sodium, total calcium, chloride, glucose, pH, $PCO_2$, albumin infusion, norepinephrine, vasopressin, enteral nutrition, IMV after AKI.

Group 1, low level; Group 2, medium level; Group 3, high level.

**Table S11.** Subgroup analysis for the association between trajectory groups and 30-day mortality in sensitivity analysis.

| Trajectory | Subgroup | HR (95%CI) | *p* | P for interaction |
|---|---|---|---|---|
| | **Age** | | | |
| Group 1 | <65 years | 1.38 (0.95~2.01) | 0.094 | 0.688 |
| | ≥65 years | 1.34 (1.02~1.77) | 0.035 | |
| Group 2 | <65 years | 1.00 (Reference) | | |
| | ≥65 years | 1.00 (Reference) | | |
| Group 3 | <65 years | 1.82 (1.24~2.67) | 0.002 | <.001 |
| | ≥65 years | 0.51 (0.30~0.85) | 0.010 | |
| | **Sex** | | | |
| Group 1 | Male | 1.64 (1.22~2.20) | 0.001 | 0.043 |
| | Female | 1.28 (0.92~1.79) | 0.147 | |
| Group 2 | Male | 1.00 (Reference) | | |
| | Female | 1.00 (Reference) | | |
| Group 3 | Male | 1.66 (1.15~2.40) | 0.006 | 0.009 |
| | Female | 0.70 (0.41~1.17) | 0.173 | |
| | **Pneumonia** | | | |
| Group 1 | No | 1.64 (1.19~2.25) | 0.002 | 0.268 |
| | Yes | 1.13 (0.83~1.53) | 0.438 | |
| Group 2 | No | 1.00 (Reference) | | |
| | Yes | 1.00 (Reference) | | |
| Group 3 | No | 1.43 (0.96~2.14) | 0.081 | 0.052 |
| | Yes | 0.91 (0.58~1.43) | 0.687 | |
| | **Hypertension** | | | |
| Group 1 | No | 1.49 (1.14~1.94) | 0.004 | 0.439 |
| | Yes | 1.25 (0.84~1.87) | 0.270 | |
| Group 2 | No | 1.00 (Reference) | | |
| | Yes | 1.00 (Reference) | | |
| Group 3 | No | 1.14 (0.79~1.64) | 0.485 | 0.933 |
| | Yes | 1.15 (0.68~1.93) | 0.602 | |
| | **Diabetes** | | | |
| Group 1 | No | 1.44 (1.10~87) | 0.007 | 0.302 |
| | Yes | 1.15 (0.77~1.71) | 0.499 | |
| Group 2 | No | 1.00 (Reference) | | |
| | Yes | 1.00 (Reference) | | |
| Group 3 | No | 1.22 (0.86~1.73) | 0.275 | 0.994 |
| | Yes | 0.99 (0.57~1.72) | 0.982 | |
| | **Cirrhosis** | | | |
| Group 1 | No | 1.40 (1.08~1.81) | 0.010 | 0.633 |
| | Yes | 1.37 (0.90~2.08) | 0.137 | |
| Group 2 | No | 1.00 (Reference) | | |

| | | | | |
|---|---|---|---|---|
| | Yes | 1.00 (Reference) | | |
| Group 3 | No | 0.64 (0.36~1.13) | 0.125 | 0.024 |
| | Yes | 1.48 (1.00~2.19) | 0.051 | |

Abbreviations: HR, hazard ratio; CI, confidence interval. Subgroup analysis was adjusted for age, ICU type, SAPS II, cirrhosis, pneumonia, malignancy, COPD, MI, acute pancreatitis, heart rate, respiratory rate, $SpO_2$, WBC, platelet, hemoglobin, total bilirubin, ALT, AST, urea nitrogen, PT, PTT, sodium, total calcium, chloride, glucose, pH, $PCO_2$, albumin infusion, norepinephrine, vasopressin, enteral nutrition, IMV after AKI.

**Table S12.** Subgroup analysis for the association between trajectory groups and 90-day mortality in sensitivity analysis.

| Trajectory | Subgroup | HR (95%CI) | *p* | P for interaction |
|---|---|---|---|---|
| | **Age** | | | |
| Group 1 | <65 years | 1.54 (1.14~2.09) | 0.005 | 0.279 |
| | ≥65 years | 1.37 (1.09~1.72) | 0.007 | |
| Group 2 | <65 years | 1.00 (Reference) | | |
| | ≥65 years | 1.00 (Reference) | | |
| Group 3 | <65 years | 1.65 (1.19~2.30) | 0.003 | <.001 |
| | ≥65 years | 0.66 (0.44~0.97) | 0.034 | |
| | **Sex** | | | |
| Group 1 | Male | 1.44 (1.13~1.84) | 0.003 | 0.885 |
| | Female | 1.60 (1.21~2.12) | <.001 | |
| Group 2 | Male | 1.00 (Reference) | | |
| | Female | 1.00 (Reference) | | |
| Group 3 | Male | 1.27 (0.93~1.74) | 0.127 | 0.431 |
| | Female | 1.00 (0.67~1.51) | 0.999 | |
| | **Pneumonia** | | | |
| Group 1 | No | 1.57 (1.21~2.03) | <.001 | 0.498 |
| | Yes | 1.27 (0.98~1.65) | 0.068 | |
| Group 2 | No | 1.00 (Reference) | | |
| | Yes | 1.00 (Reference) | | |
| Group 3 | No | 1.26 (0.90~1.77) | 0.187 | 0.410 |
| | Yes | 1.10 (0.77~1.58) | 0.587 | |
| | **Hypertension** | | | |
| Group 1 | No | 1.63 (1.31~2.03) | <.001 | 0.318 |
| | Yes | 1.16 (0.83~1.62) | 0.390 | |
| Group 2 | No | 1.00 (Reference) | | |
| | Yes | 1.00 (Reference) | | |
| Group 3 | No | 1.10 (0.82~1.49) | 0.516 | 0.920 |
| | Yes | 1.29 (0.83~2.02) | 0.262 | |
| | **Diabetes** | | | |
| Group 1 | No | 1.47 (1.17~1.83) | <.001 | 0.540 |
| | Yes | 1.36 (0.99~1.87) | 0.059 | |
| Group 2 | No | 1.00 (Reference) | | |
| | Yes | 1.00 (Reference) | | |
| Group 3 | No | 1.22 (0.91~1.64) | 0.186 | 0.967 |
| | Yes | 1.03 (0.67~1.60) | 0.891 | |
| | **Cirrhosis** | | | |
| Group 1 | No | 1.55 (1.25~1.92) | <.001 | 0.850 |
| | Yes | 1.36 (0.95~1.94) | 0.097 | |
| Group 2 | No | 1.00 (Reference) | | |

| | | | | |
|---|---|---|---|---|
| | Yes | 1.00 (Reference) | | |
| Group 3 | No | 0.72 (0.46~1.12) | 0.145 | 0.025 |
| | Yes | 1.43 (1.03~1.99) | 0.033 | |

Abbreviations: HR, hazard ratio; CI, confidence interval. Subgroup analysis was adjusted for age, ICU type, SAPS II, cirrhosis, pneumonia, malignancy, COPD, MI, acute pancreatitis, heart rate, respiratory rate, $SpO_2$, WBC, platelet, hemoglobin, total bilirubin, ALT, AST, urea nitrogen, PT, PTT, sodium, total calcium, chloride, glucose, pH, $PCO_2$, albumin infusion, norepinephrine, vasopressin, enteral nutrition, IMV after AKI.

**Table S13.** Subgroup analysis for the association between trajectory groups and 180-day mortality in sensitivity analysis.

| Trajectory | Subgroup | HR (95%CI) | *p* | P for interaction |
|---|---|---|---|---|
| | **Age** | | | |
| Group 1 | <65 years | 1.60 (1.20~2.14) | 0.001 | 0.169 |
| | ≥65 years | 1.33 (1.07~1.65) | 0.010 | |
| Group 2 | <65 years | 1.00 (Reference) | | |
| | ≥65 years | 1.00 (Reference) | | |
| Group 3 | <65 years | 1.51 (1.10~2.07) | 0.011 | 0.005 |
| | ≥65 years | 0.76 (0.53~1.08) | 0.123 | |
| | **Sex** | | | |
| Group 1 | Male | 1.44 (1.15 ~1.81) | 0.002 | 0.926 |
| | Female | 1.57 (1.21~2.04) | <.001 | |
| Group 2 | Male | 1.00 (Reference) | | |
| | Female | 1.00 (Reference) | | |
| Group 3 | Male | 1.26 (0.94~1.69) | 0.118 | 0.335 |
| | Female | 1.00 (0.68~1.48) | 0.983 | |
| | **Pneumonia** | | | |
| Group 1 | No | 1.58 (1.24~2.01) | <.001 | 0.323 |
| | Yes | 1.25 (0.97~1.60) | 0.080 | |
| Group 2 | No | 1.00 (Reference) | | |
| | Yes | 1.00 (Reference) | | |
| Group 3 | No | 1.29 (0.94~1.77) | 0.115 | 0.316 |
| | Yes | 1.09 (0.78~1.54) | 0.614 | |
| | **Hypertension** | | | |
| Group 1 | No | 1.57 (1.27~1.92) | <0.01 | 0.493 |
| | Yes | 1.23 (0.89~1.70) | 0.207 | |
| Group 2 | No | 1.00 (Reference) | | |
| | Yes | 1.00 (Reference) | | |
| Group 3 | No | 1.14 (0.87~1.51) | 0.324 | 0.708 |
| | Yes | 1.19 (0.77~1.84) | 0.435 | |
| | **Diabetes** | | | |
| Group 1 | No | 1.45 (1.17~1.79) | <.001 | 0.384 |
| | Yes | 1.33 (0.99~1.79) | 0.061 | |
| Group 2 | No | 1.00 (Reference) | | |
| | Yes | 1.00 (Reference) | | |
| Group 3 | No | 1.21 (0.91~1.60) | 0.189 | 0.835 |
| | Yes | 1.05 (0.71~1.56) | 0.808 | |
| | **Cirrhosis** | | | |
| Group 1 | No | 1.50 (1.23~1.84) | <.001 | 0.976 |
| | Yes | 1.37 (0.98~1.93) | 0.066 | |
| Group 2 | No | 1.00 (Reference) | | |

| | | | | |
|---|---|---|---|---|
| | Yes | 1.00 (Reference) | | |
| Group 3 | No | 0.81 (0.55~1.21) | 0.304 | 0.059 |
| | Yes | 1.35 (0.98~1.84) | 0.064 | |

Abbreviations: HR, hazard ratio; CI, confidence interval. Subgroup analysis was adjusted for age, ICU type, SAPS II, cirrhosis, pneumonia, malignancy, COPD, MI, acute pancreatitis, heart rate, respiratory rate, $SpO_2$, WBC, platelet, hemoglobin, total bilirubin, ALT, AST, urea nitrogen, PT, PTT, sodium, total calcium, chloride, glucose, pH, $PCO_2$, albumin infusion, norepinephrine, vasopressin, enteral nutrition, IMV after AKI.

**Table S14.** Subgroup analysis for the association between trajectory groups and 365-day mortality in sensitivity analysis.

| Trajectory | Subgroup | HR (95%CI) | *p* | P for interaction |
|---|---|---|---|---|
| | **Age** | | | |
| Group 1 | <65 years | 1.56 (1.20~2.04) | 0.001 | 0.305 |
| | ≥65 years | 1.34 (1.09~1.65) | 0.005 | |
| Group 2 | <65 years | 1.00 (Reference) | | |
| | ≥65 years | 1.00 (Reference) | | |
| Group 3 | <65 years | 1.51 (1.13~2.03) | 0.006 | 0.012 |
| | ≥65 years | 0.90 (0.65~1.25) | 0.540 | |
| | **Sex** | | | |
| Group 1 | Male | 1.44 (1.16~1.78) | 0.001 | 0.942 |
| | Female | 1.57 (1.22~2.01) | <.001 | |
| Group 2 | Male | 1.00 (Reference) | | |
| | Female | 1.00 (Reference) | | |
| Group 3 | Male | 1.43 (1.09~1.86) | 0.009 | 0.105 |
| | Female | 0.99 (0.69~1.43) | 0.956 | |
| | **Pneumonia** | | | |
| Group 1 | No | 1.57 (1.25~1.96) | <.001 | 0.404 |
| | Yes | 1.24 (0.98~1.58) | 0.075 | |
| Group 2 | No | 1.00 (Reference) | | |
| | Yes | 1.00 (Reference) | | |
| Group 3 | No | 1.45 (1.09~1.93) | 0.010 | 0.090 |
| | Yes | 1.09 (0.78~1.52) | 0.603 | |
| | **Hypertension** | | | |
| Group 1 | No | 1.52 (1.25~1.85) | <.001 | 0.652 |
| | Yes | 1.29 (0.95~1.75) | 0.097 | |
| Group 2 | No | 1.00 (Reference) | | |
| | Yes | 1.00 (Reference) | | |
| Group 3 | No | 1.20 (0.93~1.56) | 0.158 | 0.940 |
| | Yes | 1.37 (0.92~2.03) | 0.118 | |
| | **Diabetes** | | | |
| Group 1 | No | 1.44 (1.17~1.76) | <.001 | 0.509 |
| | Yes | 1.34 (1.01~1.78) | 0.043 | |
| Group 2 | No | 1.00 (Reference) | | |
| | Yes | 1.00 (Reference) | | |
| Group 3 | No | 1.25 (0.97~1.63) | 0.090 | 0.490 |
| | Yes | 1.21 (0.84~1.75) | 0.301 | |
| | **Cirrhosis** | | | |
| Group 1 | No | 1.54 (1.28~1.87) | <.001 | 0.488 |
| | Yes | 1.26 (0.91~1.74) | 0.170 | |
| Group 2 | No | 1.00 (Reference) | | |

| | | | | |
|---|---|---|---|---|
| | Yes | 1.00 (Reference) | | |
| Group 3 | No | 1.01 (0.72~1.43) | 0.948 | 0.237 |
| | Yes | 1.37 (1.02~1.83) | 0.038 | |

Abbreviations: HR, hazard ratio; CI, confidence interval. Subgroup analysis was adjusted for age, ICU type, SAPS II, cirrhosis, pneumonia, malignancy, COPD, MI, acute pancreatitis, heart rate, respiratory rate, $SpO_2$, WBC, platelet, hemoglobin, total bilirubin, ALT, AST, urea nitrogen, PT, PTT, sodium, total calcium, chloride, glucose, pH, $PCO_2$, albumin infusion, norepinephrine, vasopressin, enteral nutrition, IMV after AKI.

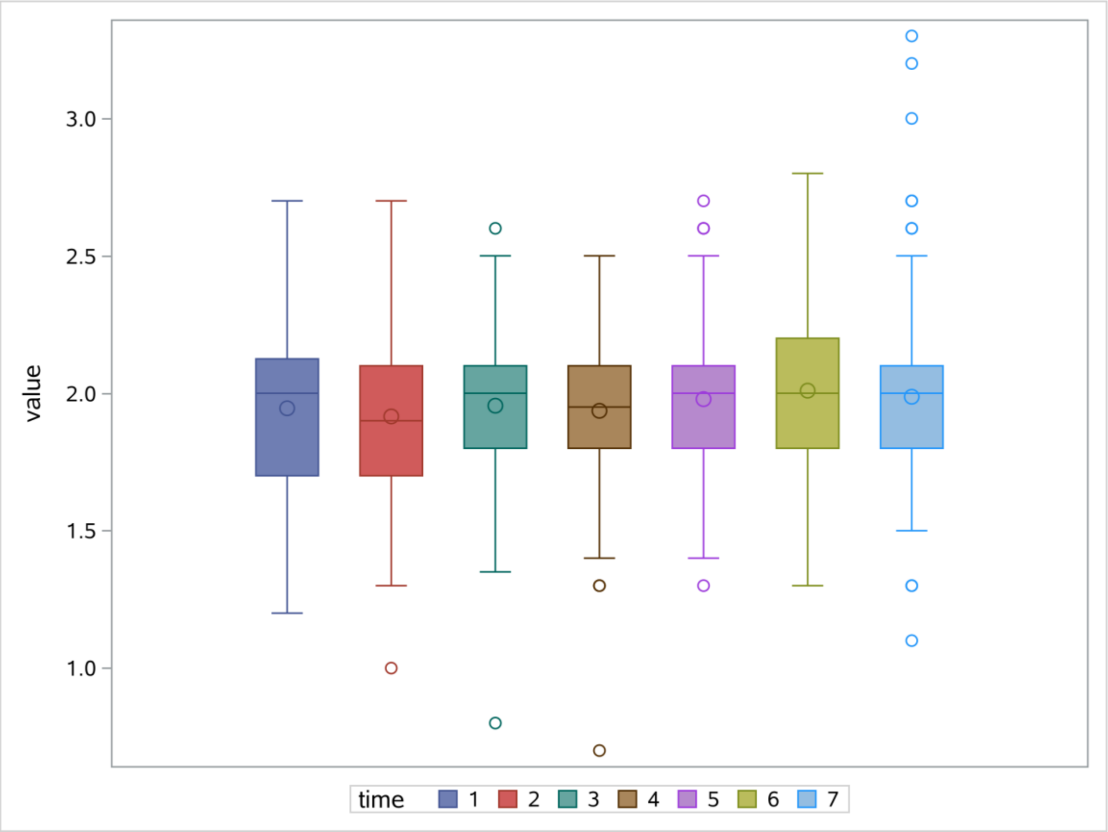


Figure S1. Distribution characteristics of serum albumin values within seven days after SA-AKI onset in patients with trajectory at a level of 2.0g/dL. The rectangle represents the interquartile range. The horizontal line in the rectangle represents the median. The circle in the rectangle represents the mean value. The remaining circles represent some scattered extreme values.

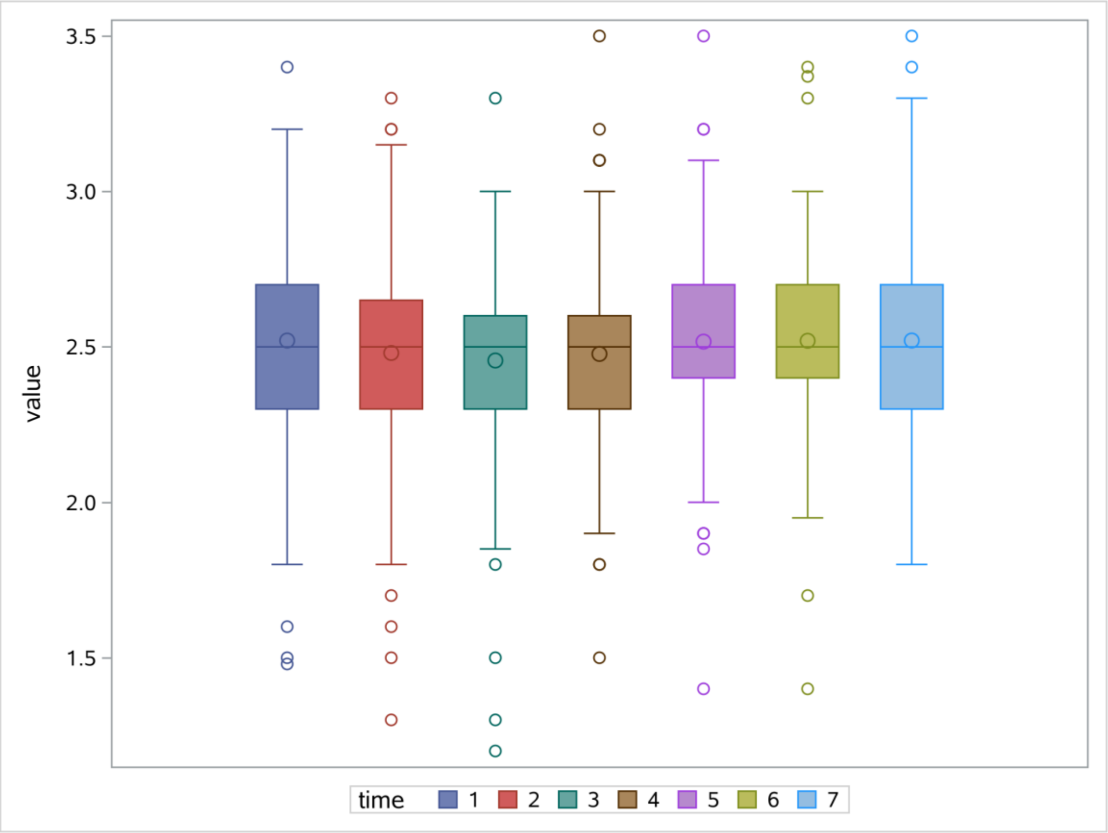


Figure S2. Distribution characteristics of serum albumin values within seven days after SA-AKI onset in patients with trajectory at a level of 2.5g/dL. The rectangle represents the interquartile range. The horizontal line in the rectangle represents the median. The circle in the rectangle represents the mean value. The remaining circles represent some scattered extreme values.

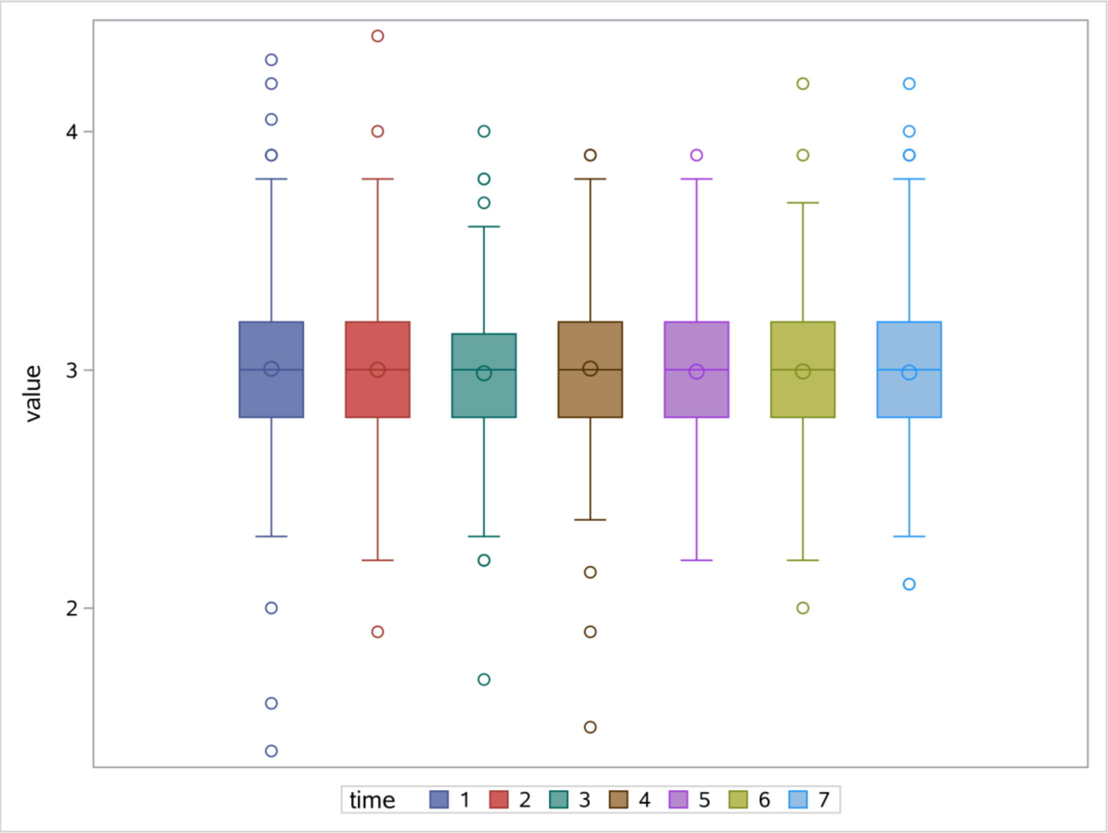


Figure S3. Distribution characteristics of serum albumin values within seven days after SA-AKI onset in patients with trajectory at a level of 3.0g/dL. The rectangle represents the interquartile range. The horizontal line in the rectangle represents the median. The circle in the rectangle represents the mean value. The remaining circles represent some scattered extreme values.

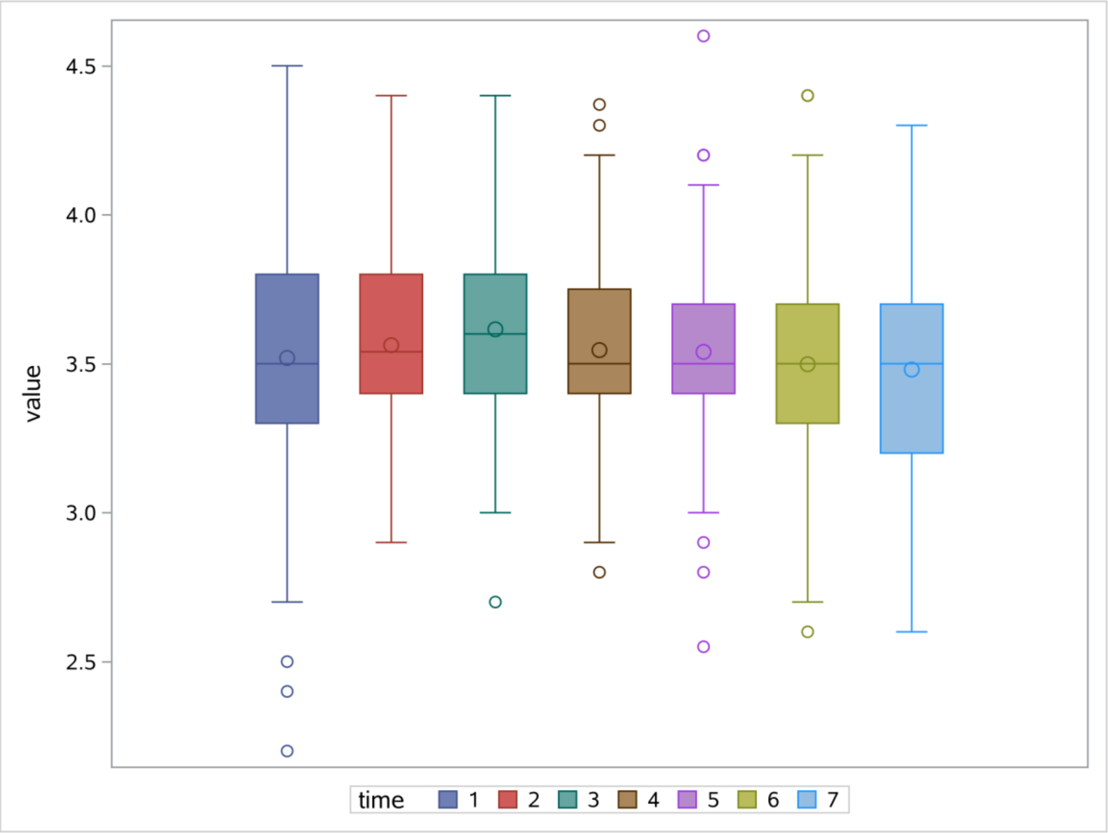


Figure S4. Distribution characteristics of serum albumin values within seven days after SA-AKI onset in patients with trajectory at a level of 3.5g/dL. The rectangle represents the interquartile range. The horizontal line in the rectangle represents the median. The circle in the rectangle represents the mean value. The remaining circles represent some scattered extreme values.

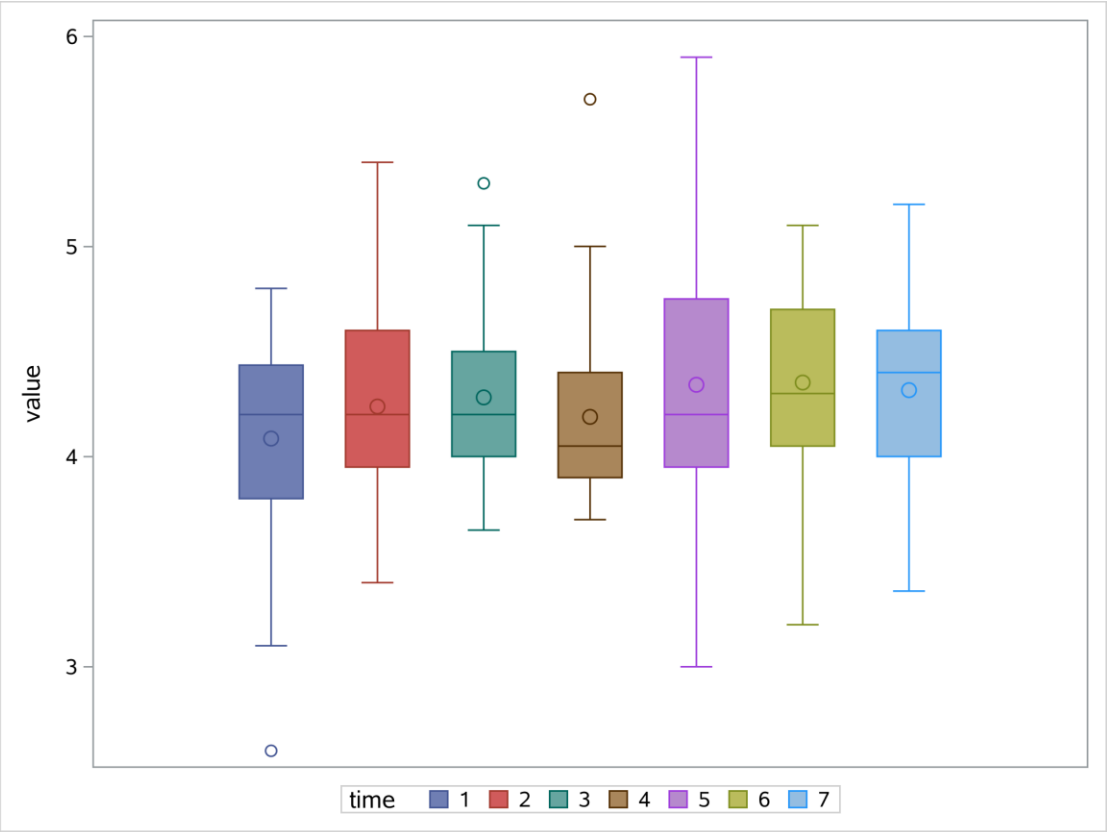


Figure S5. Distribution characteristics of serum albumin values within seven days after SA-AKI onset in patients with trajectory at a level of >4.1g/dL. The rectangle represents the interquartile range. The horizontal line in the rectangle represents the median. The circle in the rectangle represents the mean value. The remaining circles represent some scattered extreme values.

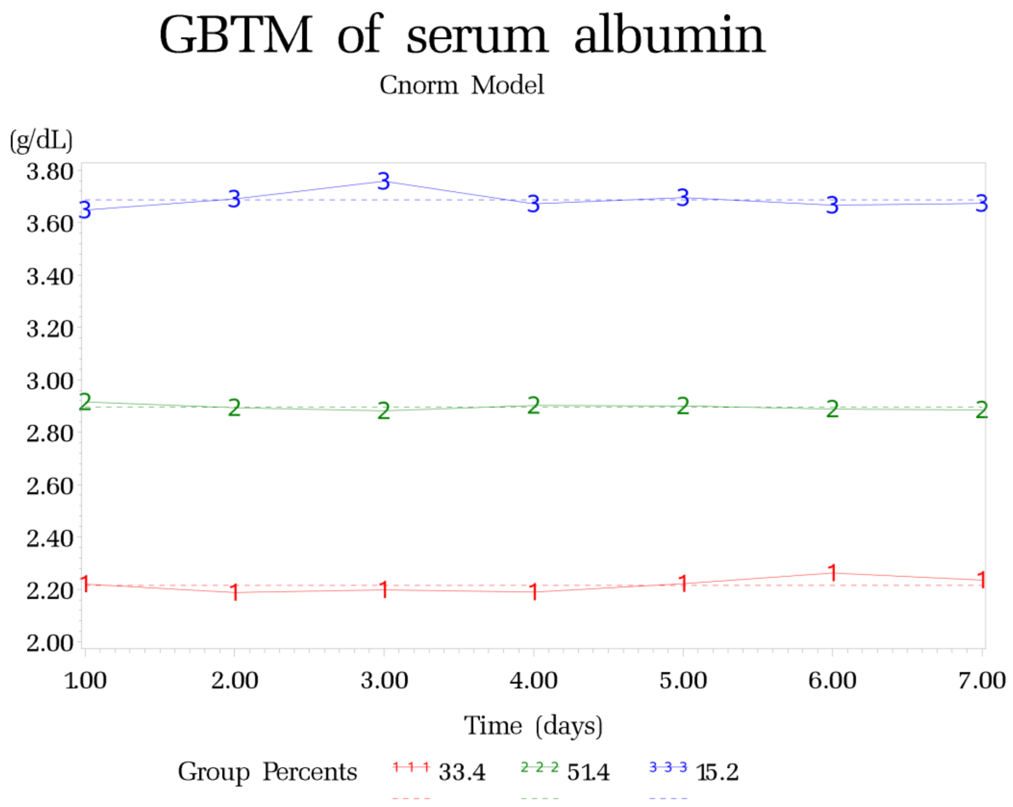


Figure S6. Trajectories of serum albumin in sensitivity analysis. GBTM, group-based trajectory modeling. The solid line represents the actual trend. The dotted line represents the predicted trend.

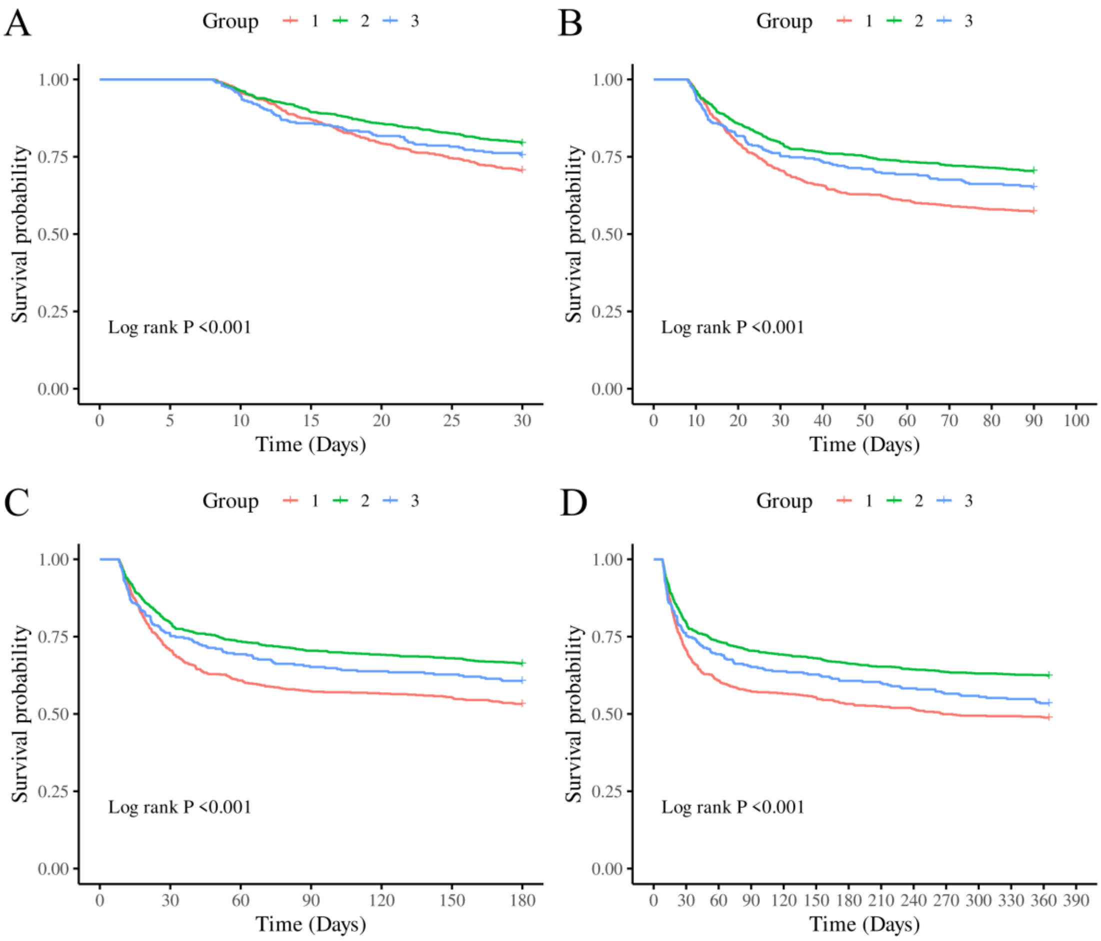


Figure S7. Kaplan-Meier survival curves for 30-day (A), 90-day (B), 180-day (C) and 365-day (D) mortality in sensitivity analysis. Group 1, low level; Group 2, medium level; Group 3, high level.
